# Math skills and microstructure of the middle longitudinal fasciculus: A developmental investigation

**DOI:** 10.1371/journal.pone.0324802

**Published:** 2025-06-11

**Authors:** Irina Buianova, Asya Istomina, Andrei Manzhurtsev, Maxim Ublinskiy, Victor Karpychev, Marie Arsalidou

**Affiliations:** 1 Department of Psychology, University of Otago, Dunedin, New Zealand; 2 Center for Language and Brain, HSE University, Moscow, Russian Federation; 3 Laboratory for Cognitive Research, HSE University, Moscow, Russian Federation; 4 Clinical and Research Institute of Emergency Pediatric Surgery and Trauma, Moscow, Russian Federation; 5 Institute of Neuroradiology, University Hospital Frankfurt, Goethe University, Frankfurt am Main, Germany; 6 Department of Neurology, School of Medicine, Emory University, Atlanta, Georgia, United States of America; 7 Department of Psychology, York University, Toronto, Canada; 8 NeuroPsyLab.com, Toronto, Canada; University of Calgary, CANADA

## Abstract

Functional neuroimaging studies have identified distributed brain networks involved in arithmetic problem-solving. However, it is still poorly understood to what extent microstructural properties of the underlying white matter contribute to mathematical performance. We investigate microstructural characteristics of one of the least studied white matter tracts, the bilateral middle longitudinal fasciculus (MdLF), reconstructed from diffusion-weighted MRI data, and their relations with mathematical performance in arithmetic tasks of varying complexity, in 56 individuals aged 10–29 years (22 children: 10–13 years; 20 adolescents: 14–17 years; 14 adults: 18–29 years). We identify group differences in math performance and diffusivity measures. We highlight linear relations with age in left fractional anisotropy and right radial diffusivity, which can serve as developmental markers. Further, we document for the first time that diffusivity values in the right MdLF are significantly lower than in the left MdLF for all groups, suggesting hemispheric asymmetry. Importantly, associations between math performance in the right MdLF favoured easier tasks and in the left MdLF favoured harder tasks. This finding is a deviation from the classic hemisphere dominance hypothesis. We propose that the observed patterns may be explained by the right-left-right hemispheric dominance hypothesis proposed by a theory of cognitive development. Our results provide new insights into the microstructural properties of the MdLF and their role in mathematical ability, with implications for understanding brain-behaviour relations.

## Introduction

**Fig 1:**
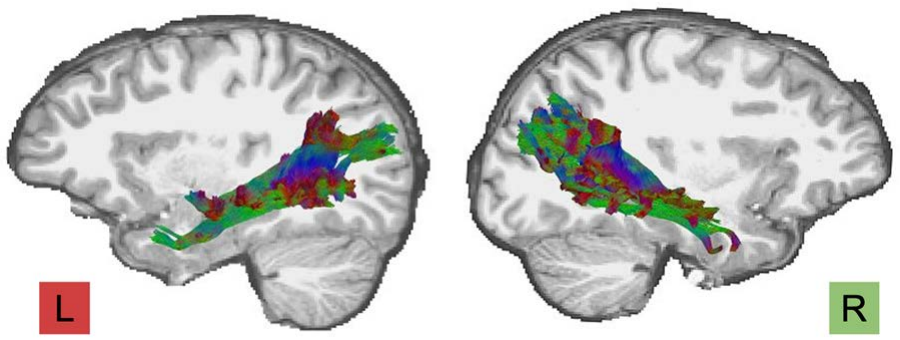
Illustration of the sagittal view of the left and right middle longitudinal fascicles on a T1 template in an adult participant obtained in the current study.

In the era of data and information, mathematics serves as the foundation for many fields, and proficiency in math is often a prerequisite for advanced studies. Neurofunctional correlates of mathematical processes are widely studied using neuroimaging and electrophysiological techniques. These include task-based functional magnetic resonance imaging (tfMRI) [1–3], functional near-infrared spectroscopy [4–7], and electroencephalography [8–10]. Fewer studies examine neurostructural correlates of math and arithmetic [11–13]. The most popular method for assessing fibre tract microstructure integrity is Diffusion Tensor Imaging (DTI). Extant DTI literature identifies links of math abilities and microstructural characteristics of inferior fronto-occipital fasciculus (IFOF) [14–16], superior longitudinal fasciculus (SLF) [16–18], inferior longitudinal fasciculus (ILF) [14,16], corona radiata [19], and corpus callosum [20]. A less-known fibre tract that connects parietal, temporal, and occipital regions is the middle longitudinal fasciculus (MdLF, Fig 1) that is linked to cognitive functions, primarily language [21–23]. The presence of the MdLF in the human brain was first documented by Makris et al. [23], and its morphology and function are the subject of ongoing research [23,24]. Although reports on the connectivity pattern of the MdLF are controversial, resection and tractography studies show that it primarily links the superior parietal lobule and, to a lesser extent, the angular and supramarginal gyri of the inferior parietal lobule, and the lateral (extrastriate) occipital cortex and cuneus, to the superior temporal gyrus, superior temporal sulcus, and temporal pole [22–25]. Notably, tfMRI identifies that many of these regions are also implicated in math [2,26–28], and one DTI study showed the relation of the MdLF microstructure to addition problems [29]. As past investigations on white matter correlates of math problem solving do not distinguish the effects of age, task difficulty, math operation or hemisphere, we explore for the first time DTI bilateral metrics in MdLF and their relations with performance in solving arithmetic operations of varying complexity in children, adolescents, and adults.

### Diffusion tensor imaging

Methods for assessing myelination range from *in-vitro* staining procedures (e.g., [30]) to non-invasive *in-vivo* magnetic resonance imaging (MRI) sequences that include diffusion-weighted imaging (dwMRI), myelin water imaging [31,32], magnetization transfer imaging [33], *g*-ratio imaging [34], and myelin mapping [35] described in detail in Buyanova & Arsalidou [36]. DTI is an application of dwMRI, a neuroimaging technique that enables the evaluation of water diffusion directionality in the axons. While advanced techniques like constrained spherical deconvolution which, unlike DTI, allows streamline propagation in more than one direction [37], are emerging, DTI remains the most widely used method to study the microstructure and orientation of myelinated white matter fibres [38–40] and their relations with cognitive functions, including mathematical abilities [36,41,42]. Fitting the diffusion tensor model to dwMRI data allows us to reconstruct white matter pathways and derive quantitative measures of axonal microstructure and integrity by estimating local fibre trajectories and orientations [38,43,44]. Diffusion metrics obtained from DTI reflect the magnitude of water diffusion in a given direction within each voxel. They are derived from a DTI matrix comprised of three eigenvalues representing one parallel (λ_1_) and two perpendicular (λ_2_ and λ_3_) directions of the diffusion tensor. Axial diffusivity (AD) measures the amount of water diffusion parallel to a white matter tract within each voxel (λ_║_ = λ _1_ > λ_2_). Radial diffusivity (RD) reflects the magnitude of water diffusion perpendicular to the tract (λ_┴_ = (λ_2_ + λ_3_)/2), and mean diffusivity (MD) reflects directionality invariant mean diffusion, i.e., diffusion in all three directions ((λ_1_ + λ_2_ + λ_3_)/3) [40,42]. Fractional anisotropy (FA) is a relative measure of directionality or the anisotropy of water diffusion along the principal direction. FA values range from 0 to 1, with higher values corresponding to highly structured tissues, such as myelinated fibres in which diffusion is facilitated in the direction parallel to the fibre orientation and restricted in the directions perpendicular to the fibres. While the three diffusivity metrics, AD, RD, and MD, are defined by the diffusion coefficient itself, FA is an integral and relative measure that is influenced by the extent to which parallel and perpendicular water movements are restricted (e.g., FA increases as RD and MD decrease) [45–48], with high and low FA indicating the presence and absence of a dominant fibre direction in a voxel, respectively. Among the factors that restrict water diffusion are fibre diameter and density, myelination, membrane permeability, and the spatial organization of the fibres (direction, coherence, and the presence of crossing or kissing fibres) [49,50]. Axonal membranes are thought to be the main contributor to the diffusion with myelination playing a secondary role [50,51]. Together, the four diffusion indices characterize different aspects of white matter microstructure, although none of them is a direct and unambiguous indicator of fibre myelination and density. Therefore, the unitarization of all four indices is advantageous for a better understanding of fibre characteristics, which we are using in the present study.

### White matter tracts and mathematical abilities

The relations between white matter tracts connecting the frontal and parietal cortical regions and mathematical abilities have been reported for the SLF, IFOF, ILF, and the corpus callosum [15,52,53]. Specifically, previous research has demonstrated a positive association between math performance and microstructural integrity of the left SLF, which connects frontal, parietal, and temporal regions [11,16,17,53,54]. Tsang and colleagues found a positive correlation between FA in the anterior SLF and approximate addition task performance in typically developing children aged 7–9 years, indicating the tract’s role in early arithmetic abilities [17]. Similarly, Lebel et al. showed that FA in the left parietal SLF was positively related to math scores in 8–9 year old children with developmental dyscalculia [18].

The ability to solve arithmetic problems is also associated with white matter connecting parietal, temporal, and occipital regions [12,14–16,55]. Rykhlevskaia and colleagues found that 7–9 year old children with developmental dyscalculia had lower FA values in the right IFOF and ILF compared to typically developing children, suggesting a link between temporoparietal and frontoparietal white matter in arithmetic skills [14]. Li et al. reported a positive association between FA in the left IFOF, the SLF, and the ILF with arithmetic performance in typically developing children aged 9–11 years [53]. Another study showed a link between the microstructure of the bilateral IFOF, numerical reasoning, and reading [15]. These associations may reflect common neural mechanisms as they were not specific to any of the tasks. Finally, the relation between white matter microstructure and math has been shown for the corpus callosum, the most extensive white matter fibre system in the human brain [26,51,56–59].

### Middle longitudinal fasciculus

The role of the MdLF in arithmetic operations remains underinvestigated. Resection and tractography studies show that MdLF primarily links the superior parietal lobule and, to a lesser extent, the angular and supramarginal gyri of the inferior parietal lobule, and the lateral (extrastriate) occipital cortex and cuneus, to the superior temporal gyrus, superior temporal sulcus, and temporal pole, albeit final consensus on its boundaries has not been reached [22–25]. Key anatomical areas that characterize the MdLF are crucial for mathematical problem solving as identified using tfMRI [60,61], however, DTI studies on MdLF are lacking. Extant research chiefly relates the MdLF to language and reading, auditory processing and speech perception [23,62–68]. Beyond language, it is thought to participate in other higher-level cognitive processes, such as the integration of audio and visual information [69], visual recognition, spatial attention, executive function, and emotion [22,70,71]. In adults, a single study suggests that the MdLF plays a role in solving addition problems and supports phonological and semantic processes during simple calculations [29]. Critically, the extent to which the MdLF relates to difficult addition problems or other arithmetic operations is unknown, as no evidence exists on the relation between MdLF metrics and mathematical ability across development.

We focus on the MdLF because it connects cortical areas critical for the processing of numerical information in children [2,27,72–75] and adults [28,76–79], as evidenced by tfMRI. Among those are temporal and occipital areas as well as parietal brain regions [2,27,72–79]. The angular gyrus, one of the terminations of the MdLF in the parietal cortex, is proposed to mediate attention [80], saccade production [81], and goal-directed salience representations [82], and is pivotal for number representation and verbal retrieval [83]. It may be non-specifically involved in general mathematical principles processing and problem solving via the regulation of visual-spatial attention and visual-spatial fact retrieval [84]. The left supramarginal gyrus, the other termination of the MdLF, supports the formation of semantic associations and phonological encoding of arithmetic facts in memory essential for mathematical conceptual understanding much like other forms of conceptual knowledge [82–87]. Broadly speaking, its role in arithmetic fact retrieval may be attributed to a mature calculation mechanism that includes semantic memory and processing [85]. Another hub of the semantic network is the left middle and superior temporal cortices. They serve as the repository for the retention of arithmetic facts within the framework of long-term memory [3], helping us understand and use math concepts [88]. With age, the representation of the facts strengthens within long-term memory, consequently facilitating the more automatic retrieval of such information with reduced cognitive effort [89]. This is in agreement with studies showing that damage to the semantic network affects how people process math, demonstrating that conceptual understanding in mathematics (e.g., mathematical reasoning and problem-solving) prominently engages the semantic network [90–94].

The inferior parietal lobule is known to be differently involved in mathematical cognition across hemispheres. The right inferior parietal lobule favours processing of complex effort-demanding, i.e., non-automatized number tasks, whereas the activity in the left inferior parietal lobule favours “active” calculation involving transformations and operations that are known to the problem solver [60,95]. This functional asymmetry is reflected in the rote-memory retrieval hypothesis that attributes easy math problem solving to the left hemisphere, consistent with old notions about hemispheric roles that attribute spatial processing to the right hemisphere and verbal processing to the left hemisphere [96]. We refer to this as the material-specific hypothesis because it relies on the type of material (i.e., verbal or spatial). An alternative hypothesis regarding hemispheric roles is given by a constructivist theory of cognitive development proposed by Pascual Leone and colleagues [60,97]. This is called the right-left-right hemispheric hypothesis which proposes that hemispheric implication is driven by a trade-off between familiarity and novelty, and a trade-off between the demand of the task (i.e., difficulty) and mental attentional capacity of the problem solver [60]. Thus, in the case of arithmetic operations, simple, automated tasks should be handled by the right hemisphere whereas tasks that are more difficult but within the mental attentional capacity of the problem solver should favour the left hemisphere. This hypothesis is unique as it suggests that problems with very high demand that exceed the mental attentional capacity of the problem solver would favour the right hemisphere, thus the name of this hypothesis, right-left-right.

To explore the relations of the MdLF and math, we introduce a new paradigm, the Parametric Math Task [98], which combines four main arithmetic operations – addition, subtraction, multiplication, and division – that include three levels of difficulty indexed by one-digit, two-digit, and three-digit problems. To capture critical developmental stages with unique cognitive and educational milestones, we evaluate the differences in DTI metrics and mathematical performance scores among typically developing children, adolescents, and adults. The focus on middle school-aged children targets a period when they master and become proficient in arithmetic facts [99], whereas adolescents undergo a pivotal stage in formal education when more complex and abstract scientific and mathematical concepts are introduced [100]. Finally, to quantify the unique effects of age and MdLF microstructure on mathematical performance, as well as the effect of age on the relations between MdLF and mathematics, we ran correlation analyses and built a series of linear regression models with each DTI metric (FA, MD, RD, AD) as a predictor variable, each mathematical performance score as a response variable, and age as a covariate.

As our model introduces many variables with multiple levels (Age: 3 levels, Math: four arithmetic operations by 3 levels for accuracy and reaction time, DTI: bilateral by 4 levels) we take an exploratory approach, albeit we form our hypotheses on developmental, cognitive, hemispheric, and microstructural variables based on past empirical and theoretical frameworks. Specifically, we anticipate that DTI metrics in MdLF will change with age (e.g., FA will increase whereas RD and MD will decrease with age [36]). We hypothesize that behavioural performance will be modulated by both age and difficulty such that accuracy will increase with age and decrease with difficulty, and vice versa for reaction time. Based on the right-left-right hypotheses, we expect that easy, i.e., one-digit problems will favour the right hemisphere whereas more difficult three-digit problems would favour the left hemisphere [60,97]. Lastly, we anticipate more relations between DTI metrics and performance as a function of difficulty rather than arithmetic operation.

### Methods

#### Participants.

Sixty-two healthy, right-handed participants aged 10–29 years (mean age = 15.97 ± 5.09 years; 31 females) were recruited from local schools, high schools, and universities to participate in an MRI study on the neural correlates of math performance. Participants were fluent Russian speakers and had normal or corrected-to-normal vision. None of the participants had any contraindications to MRI scanning (e.g., MRI-incompatible implants or claustrophobia), a family history of psychiatric or neurological disorders, head trauma, and drug or alcohol abuse. The study was approved by the Institutional Review Board of HSE University and was conducted in accordance with the Declaration of Helsinki. The parents of children and adult participants aged 18 years and above signed written informed consent. After the scanning session, participants received structural MRI images of their brains and a monetary reward, corresponding to about $10 for adults and $30 for parents with children. Participants with incomplete dwMRI data (*n* = 5) were excluded from tractography analysis. For one participant, the fibre tract of interest was not reconstructed because of motion artifacts. The final sample yielded 56 participants divided into three groups: 22 children (10–13 years, mean age = 11.68 ± 1.21 years; 10 females), 20 adolescents (14–17 years, mean age = 15.45 ± 1.23 years; 11 females), and 14 adults (18–29 years, mean age = 23.86 ± 2.51; 7 females).

### Experimental procedure

**Fig 2:**
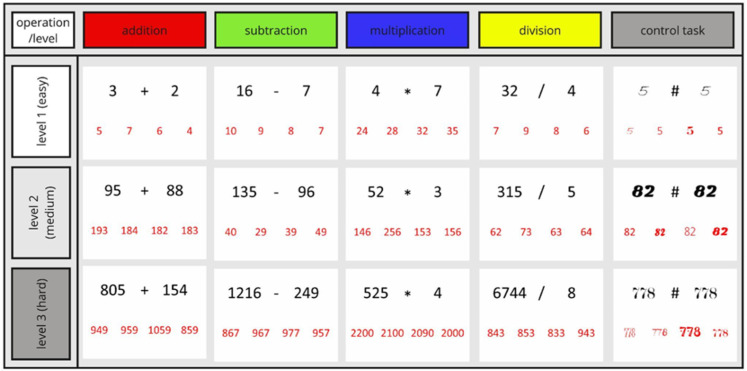
Example of arithmetic tasks and difficulty levels used in PMT. Control tasks were displayed as pairs of one-, two-, or three-digit numbers, separated by a “#” sign on the top part of the screen. Similar to the math tasks, the control tasks contained four response options. The task for the participants was to identify which number was written in the same font as the pair on the upper part of the screen. All task blocks and stimuli within blocks were randomly presented. Specifically, for each condition, 100 stimuli were created that appeared one by one on the screen in a random order. Hence, each participant was assigned a unique order of blocks and math problems.

**Fig 3:**
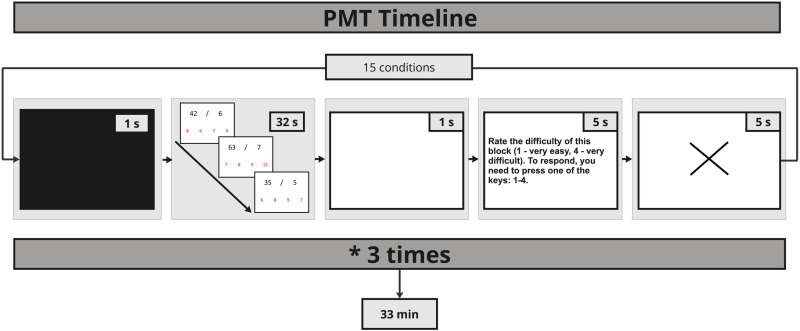
The sequence of events in the PMT. Participants first viewed a black screen for 1 second (s), followed by the main task (an arithmetic operation or control) for 32 s. Subsequently, a white screen was presented for 1 s. Participants then rated their effort for solving the task within 5 s, after which a fixation cross was displayed. This sequence was repeated for 15 iterations to complete a full run, with the entire PMT comprising 3 runs of 11 minutes (min) each. The total task time was approximately 33 min.

Math ability was assessed with the PMT [98]. The PMT includes four types of arithmetic operations – addition, subtraction, multiplication, and division and three levels of difficulty with one-, two-, and three-digit numbers, respectively. Participants were asked to solve math problems that appeared on a screen and select the correct one out of four answers displayed (Fig 2). Distractor answers contained a number that differed from the correct solution by 1–2, 10–20, or 100–200 units, depending on the difficulty level, to discourage participants from making approximate calculations. There were twelve tasks and three control conditions. Thus, with 15 conditions (12 math tasks plus 3 control tasks) each repeated three times, the experiment comprised a total of 45 blocks (Fig 3). Each condition appeared in a block that lasted for 32 seconds. During this time, participants were asked to give as many correct answers as possible. After each block, participants were asked to rate their effort to solve tasks from 1 (very easy) to 4 (very difficult), within 5 seconds, a white screen appeared after rating the response. Then a fixation cross appeared for 5 seconds before the next block. Task complexity levels which varied by the number of digits included (Fig 2):

(a) **Easy level:** addition and multiplication problems that contain two single-digit addends (or multipliers); the sum ranged from 2 to 18 and the multiplication product ranged from 4 to 81, as per the multiplication table. Subtraction and division were the reverse problems of the addition and multiplication tasks, respectively.
(b) **Medium difficulty level:** addition problems with two two-digit addends, and the sum ranged from 21 to 198. Multiplication problems contained one single-digit multiplier and one two-digit multiplier, the multiplication product ranged from 22 to 891. Subtraction and division were the reverse problems of the addition and multiplication tasks, respectively.
(c) **Difficult level:** addition problems with two three-digit addends, and the sum ranged from 201 to 1998. Multiplication problems contained one single-digit multiplier and one three-digit multiplier, the multiplication product ranges from 301 to 8991. Subtraction and division were the reverse problems of the addition and multiplication tasks, respectively.

As measures of behavioural performance for each task, we used accuracy (the proportion of correct answers) and reaction time averaged across three blocks. In this study, we do not report performance on the control task as it was only used as a comparison task in the tfMRI analysis. Behavioral data were obtained during tfMRI scanning in three runs of 11 minutes each, with a total task time of approximately 33 min. The PMT experiment was designed and executed using Presentation^®^ software (Version 23.0, Neurobehavioral Systems, Inc., Berkeley, CA), synchronized with the MRI Scanner. PMT data were gathered using two MRI-compatible button boxes, each equipped with two keys for the left and right hands. During the PMT task, participants selected the correct answer by pressing one of four buttons, labeled “1”, “2”, “3”, and “4”, after a brief training session to familiarize them with the button locations.

### MRI data acquisition and processing

The study was conducted in the MRI facility of the Clinical and Research Institute of Emergency Pediatric Surgery and Trauma. MRI data were acquired on a 3T MRI scanner (Philips Achieva dStream 3T, the Netherlands) and included anatomical T1-weighted, dwMRI, and tfMRI scans. In this study, we used only dwMRI and structural T1-weighted MRI data. The high-resolution T1-weighted images were collected with a 3D fast gradient inversion recovery sequence (TR = 2300 ms, TE = 3.7 ms, 1 mm isotropic voxel, FA = 8°; 179 slices). dwMRI data were collected using a DW-SE-EPI echo planar imaging sequence (TR = 7000 ms, TE = 72 ms, matrix size = 128 × 128, FOV = 224 × 224 mm^2^, voxel 1.75 × 1.75 × 2 mm^3^, flip angle = 90°, *b*_0_ = 0 (1 acquisition), *b* = 700 s/mm^2^, 32 non-colinear orientations of diffusion gradient, slice thickness 2 mm, 60 slices, parallel imaging SENSE factor 2, partial Fourier sampling factor 6/8). The sequence was repeated once in the anterior-to-posterior phase-encoding direction.

Participant motion, eddy current distortion correction, and whole-brain tractography were performed using the Quantitative Imaging Toolkit (QIT) software [101]. QIT is a tool for white matter reconstruction that integrates common neuroimaging analysis software, allowing the creation of customized pipelines. For motion artifact correction, QIT estimates and applies motion parameters directly to the dwMRI data. It uses *VolumeDwiMotionEstimation* to evaluate motion parameters by registering each gradient direction to a reference volume and *VolumeDwiCorrect* to align the dwMRI images and apply the motion parameters to the dwMRI data via FSL’s *eddy_correct*. White matter pathways are reconstructed from fibre orientation data in the T1 space, preserving the anatomical accuracy of the fibre tracts relative to the participant’s brain anatomy. Cortical regions are labeled based on the Desikan-Killiany (aparc) atlas, as a part of QIT pipeline. To coregister dwMRI images to a T1-weighted image, QIT uses deformable intra-subject registration, and for anatomical normalization, it applies the Talairach transformation to align the T1-weighted image to a standard space. Finally, to reconstruct white matter pathways, QIT performs reinforcement whole-brain tractography based on the hybrid probabilistic and deterministic approach [102]. The idea behind hybrid probabilistic and deterministic tractography stems from reinforcement learning: it balances probabilistic (exploration) and deterministic (exploitation) steps, allowing iterative refining of the bundles and optimal, biologically plausible assignment of fibre compartments. Reinforcement tractography includes two major steps – exploration and exploitation. In the exploration step, the algorithm applies probabilistic tractography to identify possible fibre trajectories considering the uncertainty in fibre orientation. The information obtained in the probabilistic step about the most likely pathways is then used in the exploitation step to guide deterministic (streamline) tractography to ensure the anatomical accuracy of the reconstructed pathways.

As a measure of the microstructural characteristics, we used four diffusion metrics – FA, AD, RD, and MD. Tractography analysis was partly supported by computational resources of HPC facilities at HSE University [103].

### Statistical analysis

Statistical analysis was performed in RStudio Version 2023.06.1. Plots were generated with *ggplot* in RStudio. Inferential statistics were explored using analyses of variance (ANOVA) and correlational statistics. DTI and PMT variables are tabulated in the main text and supporting information (S1 and S2 Tables in S1 File).

First, to investigate the developmental pattern of PMT performance and MdLF microstructural characteristics, we computed Pearson correlation coefficients between PMT scores, DTI metrics, and age. Additionally, we conducted a correlation analysis to evaluate the relations between DTI metrics. Next, we ran a series of one-way ANOVA analyses to assess the between-group differences in PMT performance and four DTI metrics in the MdLF (FA, AD, RD, and MD). To discern significant differences between the group means, we used Tukey’s Honestly Significant Difference (HSD) at *p* < 0.05 controlling for multiple comparisons. Further, to reveal potential interhemispheric asymmetry in MdLF microstructure, we also conducted Student’s *t*-*t*ests to compare the values of DTI metrics in the left and right hemispheres in each group. To examine relations among DTI metrics in the MdLF and PMT performance, we built a series of linear regression models where we treated each PMT performance score (accuracy and reaction time) as a response variable, each DTI metric (bilaterally) as an explanatory variable, and age as a covariate, e.g.,


Accuracy Addition Level 1 ~ age * (FA left MdLF + FA right MdLF)

Given that diffusion metrics change with age, we assume that their effect on math performance also varies with age, and therefore include an interaction term in our models to capture the dynamic nature of brain and cognitive development. We did not control for brain volume and sex because reports suggest that brain volume is stable after 9 years [104–106], and albeit our sample was balanced in terms of sex, it would be small for meaningful interpretation. To facilitate the interpretation and comparison of the effects of different explanatory variables, we standardized age and DTI metrics before fitting a model. Standardization involves transforming the explanatory variables to have a mean of zero and a standard deviation of one. This process allows the coefficients to be interpreted as the number of standard deviations the dependent variable changes for a one standard deviation change in the predictor variable. Given the different scales and units of the DTI metrics, standardization was essential to ensure that the regression coefficients, including the interaction effects, were comparable across models. Finally, to examine overall relations among all DTI metrics in the MdLF and behavioural performance, we computed bivariate Pearson’s r correlation coefficients with and without controlling for age. To control for age, we first fitted a multiple linear regression model with age and DTI or PMT performance metrics as predictors. Next, we obtained predicted values for DTI and PMT performance from the age and calculated the residuals (residuals = original values of DTI/ PMT performance metrics – values of DTI/ PMT performance metrics predicted from linear regression model).

## Results

### Correlation analysis: PMT performance and age

**Fig 4:**
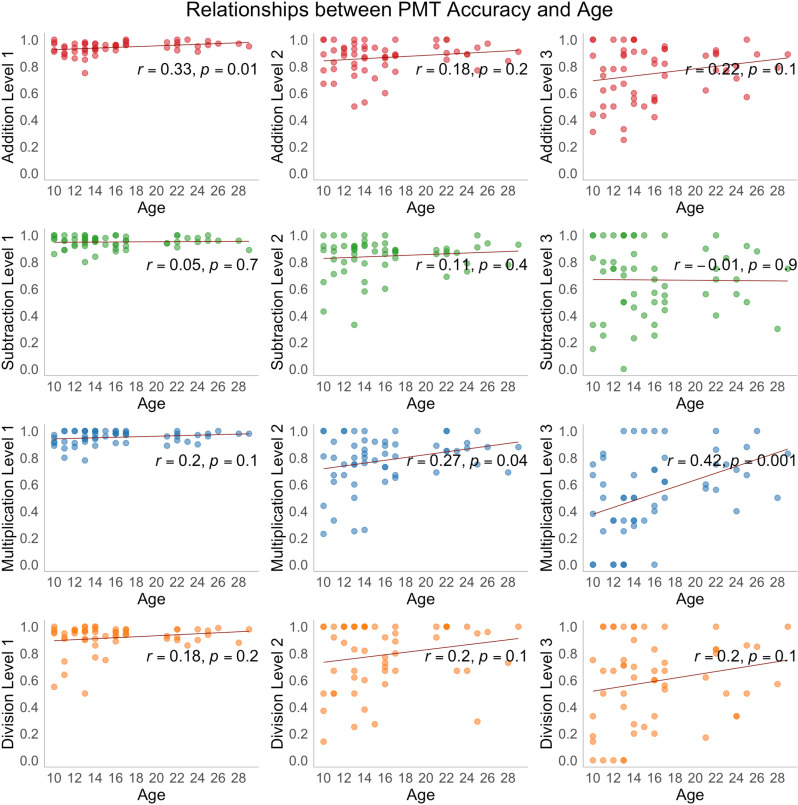
Scatterplots showing the distribution of PMT accuracy scores across three levels of addition, subtraction, multiplication, and division tasks as a function of age (years), Pearson correlation coefficients (r), and corresponding significance levels (p). The regression line represents the direction of the linear relation between PMT reaction time and age, *p*-values at < 0.05 are considered statistically significant.

**Fig 5:**
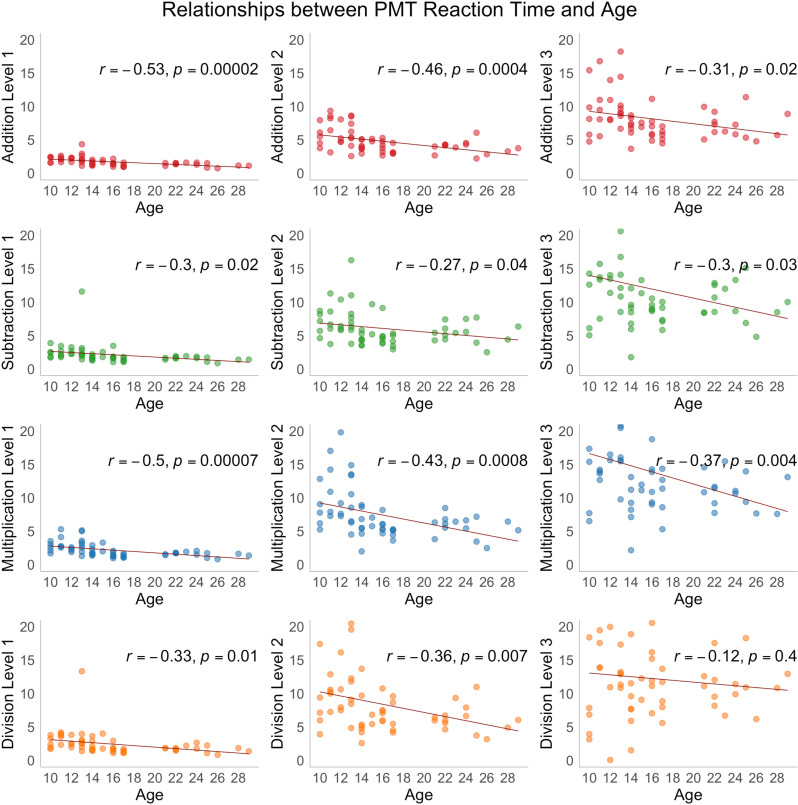
Scatterplots showing the distribution of PMT reaction time across three levels of addition, subtraction, multiplication, and division tasks as a function of age (years), Pearson correlation coefficients (r), and corresponding significance levels (p). The regression line represents the direction of the linear relation between PMT reaction time and age, *p*-values at < 0.05 are considered statistically significant.

Accuracy generally improved with age, but significant correlations between accuracy and age were observed only in a few instances – in one-digit addition and two- and three-digit multiplication (Fig 4). Reaction time in all tasks showed a negative association with age except for three-digit division (Fig 5). The raw values of the PMT performance scores for each participant are provided in S1 Table in S1 File.

### Correlation analysis: MdLF microstructure and age

Anatomically, the MdLF fibres imaged run through the superior and inferior parietal lobules and occipital cortex and terminate within the superior temporal gyrus and temporal pole (Fig 1). The raw values of the DTI metrics for each participant and correlation analysis statistics (Pearson *r* and *p*-values for correlations between DTI metrics, and DTI metrics and age) are provided in S2 and S3 Tables in S1 File.

**Fig 6:**
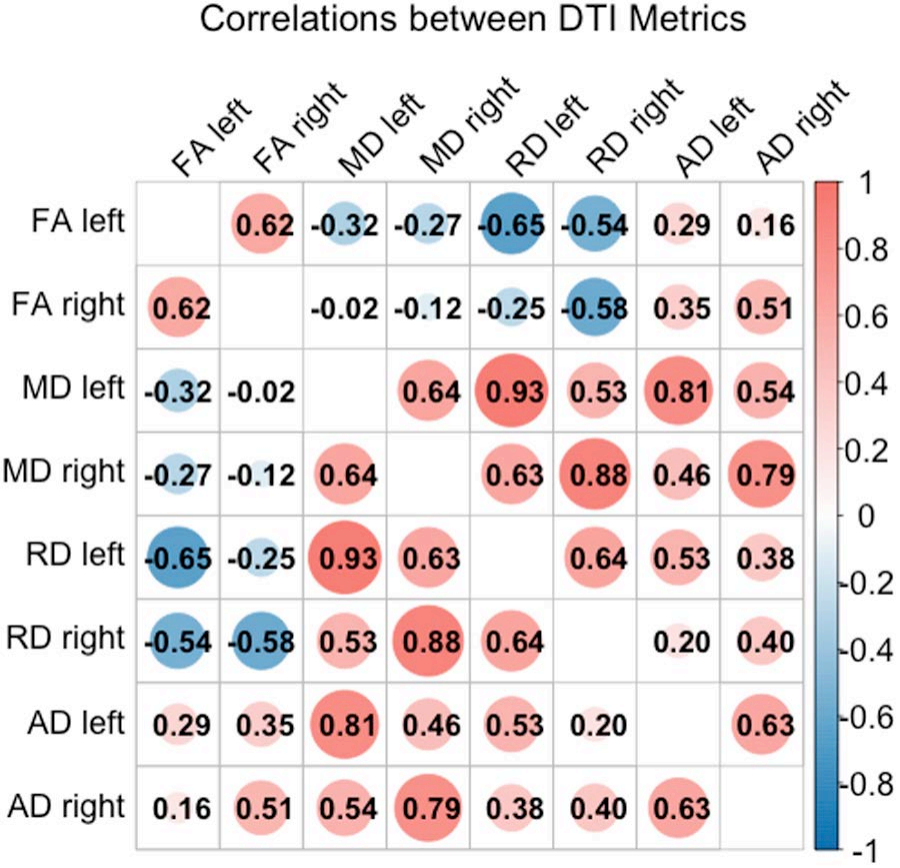
Illustration of the relations between DTI metrics Correlation matrix of Pearson *r* values for each pair of diffusion metrics. The size and colour intensity of the circles represent the magnitude and significance (*p* < 0.05) of the association. The only non-significant correlations are between FA in the right MdLF and MD bilaterally, and RD in the left MdLF; between FA in the left MdLF and AD in the right MdLF; between AD in the left MdLF and RD in the right MdLF. FA – fractional anisotropy, AD – axial diffusivity, RD – radial diffusivity, MD – mean diffusivity.

**Fig 7:**
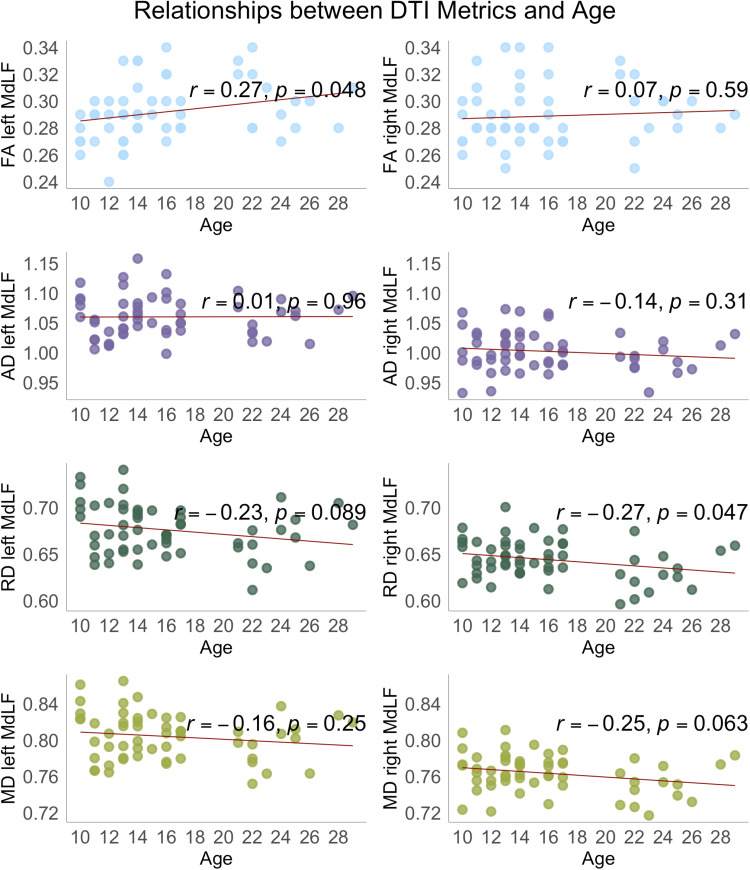
Illustration of the relations between DTI metrics and age (years). Scatterplots showing the distribution of FA, AD, RD, and MD values as a function of age, Pearson correlation coefficients (r), and corresponding significance levels (p). The regression line represents the direction of the linear relations between diffusion metrics and age. FA – fractional anisotropy, AD – axial diffusivity, RD – radial diffusivity, MD – mean diffusivity; *p*-values at < 0.05 are considered statistically significant.

There was a significant and strong positive correlation between RD and MD in each hemisphere, with Pearson *r* values ranging from 0.8 to 0.9. AD correlated positively not only with RD and MD but also with FA (Fig 6). In the left hemisphere, FA values correlated positively with age, whereas RD values correlated negatively. MD exhibited a similar, though non-significant, trend to RD (Fig 7).

### Group analysis: Behavioural results

**Fig 8:**
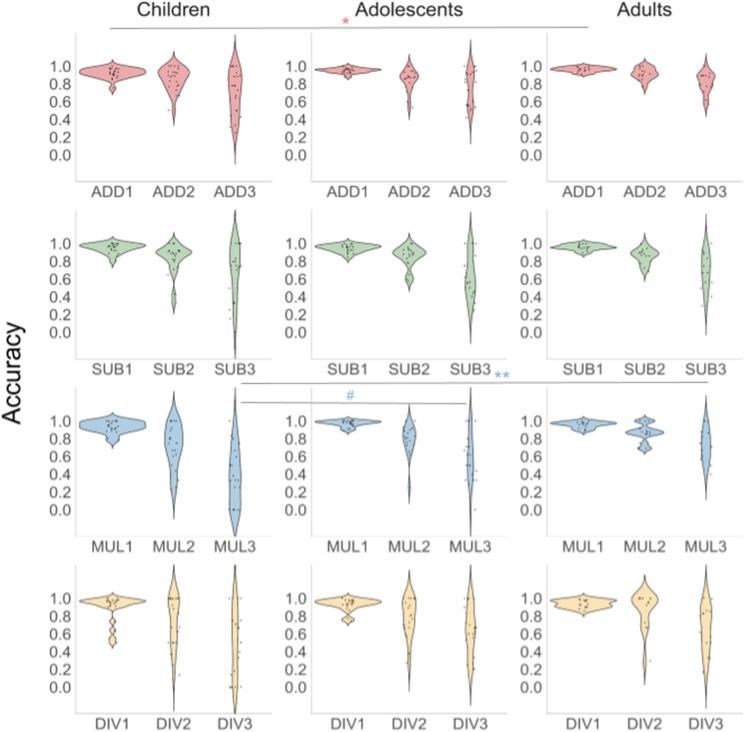
Violin plots of the values of PMT accuracy in children, adolescents, and adults. Statistically significant differences between age groups are outlined: # – *p* < 0.05, ## – *p* < 0.01, and ### – *p* < 0.001 for the differences between children and adolescents; * – *p* < 0.05, ** – *p* < 0.01, and *** – *p* < 0.001 for the differences between children and adults. The dashed line denotes differences between corresponding levels.

**Fig 9:**
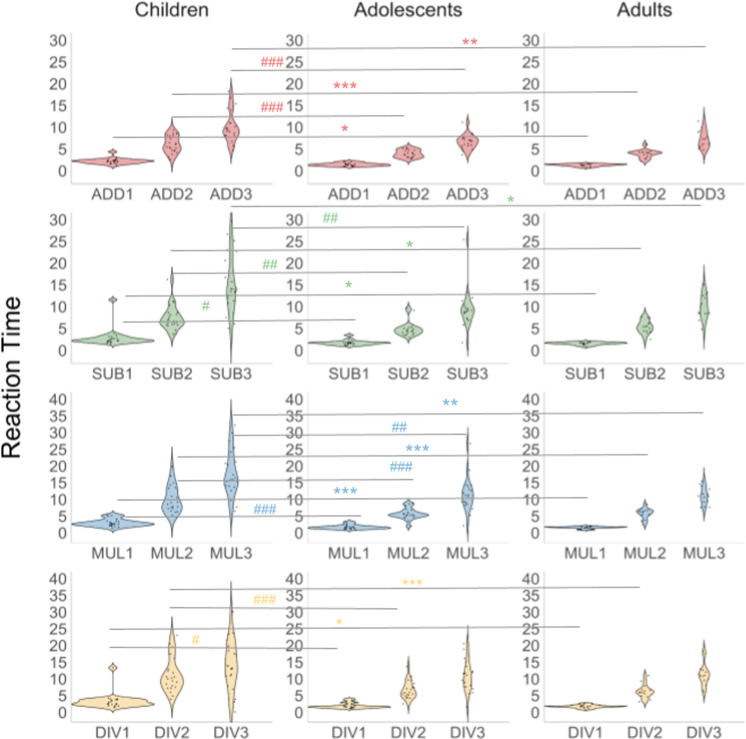
Violin plots of the values of PMT reaction time in children, adolescents, and adults. Statistically significant differences between age groups are outlined: # – *p* < 0.05, ## – *p* < 0.01, and ### – *p* < 0.001 for the differences between children and adolescents; * – *p* < 0.05, ** – *p* < 0.01, and *** – *p* < 0.001 for the differences between children and adults. The dashed line denotes differences between corresponding levels.

Descriptive statistics for behavioural results on accuracy (i.e., proportion correct) and reaction time, and the results of the ANOVA analyses are tabulated in S4–S7 Tables in S1 File. Mean accuracy scores ranged from 0.89–0.97 for Level 1, 0.72–0.92 for Level 2, and 0.36–0.81 for Level 3. Mean accuracy differed significantly between children and adults in Level 1 addition tasks (*p* < 0.05). Level 3 multiplication accuracy was also significantly different between children and adults, as well as between children and adolescents (*p* < 0.05) with children having lower overall accuracy than adolescents and adults (Fig 8). Mean reaction time ranged between 1.31–3.33 s for Level 1, 3.90–11.20 s for Level 2, and 7.2–14.24 s for Level 3. Children performed more slowly, i.e., had significantly higher mean reaction time than adolescents (*p* < 0.01) and adults (*p* < 0.001) across all arithmetic tasks, except for the Level 3 division (Fig 9).

### Group analysis: DTI results

**Fig 10:**
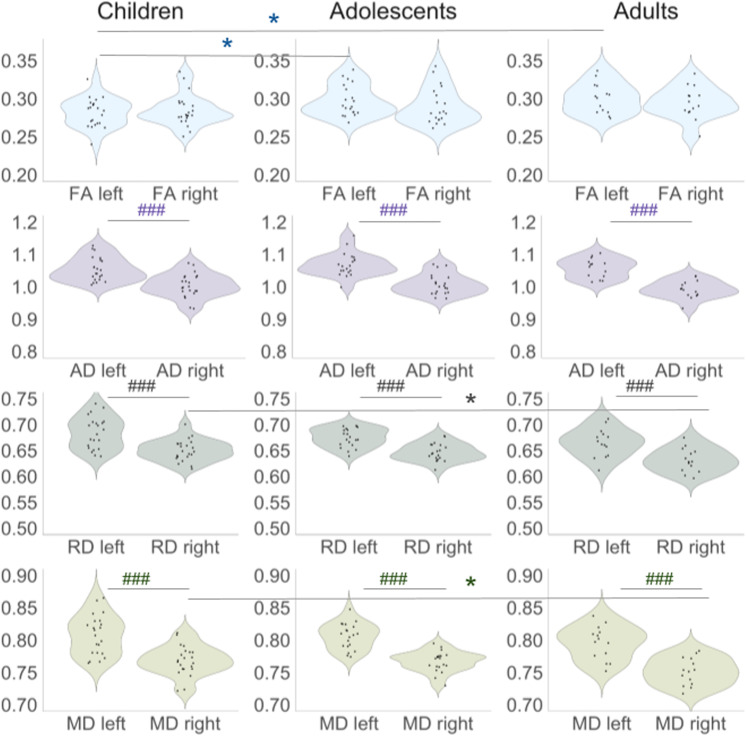
Violin plots of the values of diffusion metrics in the left and right MdLF in children, adolescents, and adults. Significant differences between age groups and the left and right hemispheres are outlined: * – *p* < 0.05 for between-group effects, ### – *p* < 0.001 for within-group effects. FA – fractional anisotropy, AD – axial diffusivity, RD – radial diffusivity, MD – mean diffusivity.

Mean, standard deviation, and corresponding between-group differences in DTI metrics are illustrated in Fig 10 and tabulated in S8 and S9 Tables in S1 File. Mean values for FA ranged between 0.282–0.301, for AD between 1.00–1.06 (×10^3^), for RD between 0.631–0.684 (×10^3^), and for MD between 0.751–0.808 (×10^3^). Significant age-related differences are found between children and adolescents (children < adolescents, *p* < 0.05), and children and adults (children < adults, *p* < 0.05) in the mean values of FA in the left MdLF, as well as RD (children > adults, *p* < 0.05) and MD (children > adults, *p* < 0.05) in the right MdLF (Fig 4 and Fig 5). Effects of hemisphere showed that values of the AD, RD, and MD in the left MdLF were significantly higher than in the right MdLF in all groups (*p* < 0.001; Fig 10 and S10 Table in S1 File).

### Linear regression model: DTI metrics and accuracy

Higher FA values in the right hemisphere were associated with lower accuracy in the Level 1 division task (Standardized *β* = −1.30, *p* = 0.030). Lower AD in the right hemisphere predicted higher accuracy in the Level 1 (Standardized *β* = −1.54, *p* = 0.008) and Level 2 (Standardized *β* = −1.59, *p* = 0.006) division tasks. However, this effect decreased with age, as indicated by a significant interaction between age and AD in a linear regression model (Standardized *β* = 1.40, *p* = 0.025 for Level 1 and Standardized *β* = 1.57, *p* = 0.011 for Level 2). Lower MD (Standardized *β* = −1.58, *p* = 0.005) and, marginally, RD (Standardized *β* = −1.22, *p *= 0.058) in the right hemisphere were also associated with better accuracy in the Level 2 division task, and these associations diminished with age (Standardized *β* = 1.57, *p* = 0.007 for MD and Standardized *β* = 1.27, *p* = 0.049 for RD). Finally, a model based on MD revealed a significant main effect of MD in the right MdLF on accuracy in the Level 2 multiplication task (Standardized *β* = −1.12, *p* = 0.048; Table 1).

**Table 1:** Statistically significant effects of DTI metrics and age on PMT accuracy.

Model		Standardized *β*	Standard Error	*t*	*t*(*p*)	*R* ^*2*^	Adjusted *R*^*2*^	*F*	*F*(*p*)
DIV1 ~ FA	FA right	−1.34	0.060	−2.24	0.030	0.18	0.09	2.13	0.077
DIV1 ~ AD	AD right	−1.54	0.058	−2.75	0.008	0.18	0.10	2.19	0.070
	age:AD right	1.40	0.004	2.31	0.025				
DIV2 ~ AD	AD right	−1.59	0.133	−2.90	0.006	0.21	0.14	2.73	0.030
	age:AD right	1.57	0.009	2.65	0.011				
DIV2 ~ RD	*RD right*	*−1.22*	*0.154*	*−1.94*	*0.058*	0.13	0.05	1.55	0.191
	age:RD right	1.27	0.009	2.02	0.049				
MUL2 ~ MD	MD right	−1.12	0.108	−2.03	0.048	0.19	0.11	2.29	0.059
DIV2 ~ MD	MD right	−1.58	0.132	−2.91	0.005	0.21	0.13	2.63	0.034
	age:MD right	1.57	0.008	2.84	0.007				

DIV – division, MUL – multiplication, FA – fractional anisotropy, AD – axial diffusivity, RD – radial diffusivity, MD – mean diffusivity, 1 and 2 correspond to difficulty levels 1 and 2. The complete results of the linear regression models are provided in the supporting information (S11 and S12 Tables in S1 File). Marginally significant relations are highlighted in italics.

The main effect of age on accuracy was statistically significant in Level 1 addition and Levels 2 and 3 multiplication tasks.

### Linear regression model: DTI metrics and reaction time

We found a significant negative relations between AD in the left MdLF and reaction time in the Level 3 division task (Standardized *β* = −1.30, *p* = 0.04). Similarly, negative relations were observed between reaction time in the Level 3 division tasks and RD (Standardized *β* = −1.61, *p* = 0.005) and MD (Standardized *β* = −1.65, p = 0.004) in the left MdLF. For both RD and MD, the negative association between DTI metrics and PMT performance decreased with age, as indicated by a significant positive interactions (Standardized *β* = 1.35, *p* = 0.014 for RD and Standardized *β* = 1.40, *p* = 0.013 for MD). Finally, there was a significant main effect of MD in the right MdLF on reaction time in Level 3 subtraction tasks (Standardized *β* = −1.14, *p* = 0.044; Table 2).

**Table 2:** Statistically significant effects of DTI metrics and age on PMT reaction time.

Model		Standardized *β*	Standard Error	*t*	*t*(*p*)	*R* ^*2*^	Adjusted *R*^*2*^	*F*	*F*(*p*)
RT DIV3 ~ AD	AD left	−1.30	3.57	−2.11	0.040	0.12	0.03	1.35	0.26
RT DIV3 ~ RD	RD left	−1.61	3.14	−2.97	0.005	0.18	0.10	2.22	0.07
	age:RD left	1.35	0.19	2.55	0.014				
RT SUB3 ~ MD	MD right	−1.14	3.20	−2.07	0.044	0.18	0.10	2.26	0.06
RT DIV3 ~ MD	MD left	−1.65	3.13	−3.06	0.004	0.19	0.11	2.31	0.06
	age:MD left	1.40	0.19	2.59	0.013				

DIV – division, AD – axial diffusivity, RD – radial diffusivity, MD – mean diffusivity, 3 corresponds to difficulty level 3. The complete results of the linear regression models are given in the supporting information (S11 and S12 Tables in S1 File).

The main effect of age on reaction time was statistically significant in all addition and multiplication tasks, Levels 1 and 3 subtraction tasks, and Levels 1 and 3 division tasks.

Because the main effects of age on PMT performance scores (accuracy and reaction time) observed in regression analysis agree with the results of the correlation analysis reported above, we provide full summary statistics for each linear regression model, including non-significant effects, in supporting information (S11 and S12 Tables in S1 File). The results of correlations analyses with and without controlling for age are outlined in supporting information (S13 and S14 Tables in S1 File).

## Discussion

We examined microstructural indices in the MdLF and their association with scores on arithmetic problems of three levels of complexity, considering hemisphere and age as factors of variation. We discuss key findings based on math scores, fibre tract metrics, and their relations in the context of cognitive and brain development theories. (a) Math scores show that generally, accuracy decreases and reaction time increases as a function of task difficulty, and reaction time decreases as a function of age, supporting our expectations. Reaction time shows more significant correlations with age as a function of task and difficulty. Significant between-group differences are observed mainly for reaction time. As this was a self-paced task, findings confirm the effect of difficulty and suggest that generally, participants slowed down to attain accuracy at higher difficulty levels. (b) DTI metrics RD and MD decrease and FA increases with age, as we hypothesized based on past empirically demonstrated developmental patterns of other association white matter tracts [30,107–110]. In agreement with our hypothesis, FA tended to increase and RD and MD decreased with age, as revealed by significant correlations. Specifically, we expand on past findings showing relations with age in left FA, and RD, and, marginally MD in the right hemisphere, suggesting that these metrics may be better markers for linear age effects in the MdLF. An exploratory finding we document for the first time is that AD, RD, and MD values in the right MdLF are significantly lower than in the left MdLF for all groups, suggesting hemispheric asymmetry. (c) DTI metrics in MdLF are associated with performance on math tasks, favouring the right hemisphere for accuracy mainly for easier levels (1 and 2) and the left hemisphere for reaction time mainly for harder levels (2 and 3). This suggests that the right and left MdLF contribute to the shared variance of microstructure indices and math ability. Importantly, this is the first study to document that the MdLF in both hemispheres is linked with mathematical performance in easy and difficult tasks. Critically, our results show that more difficult tasks, which are less likely to rely on rote memory retrieval (i.e., storing of information in long-term memory through repetition) [96], yield more associations in the left hemisphere, whereas tasks that are easier yield more associations in the right hemisphere. This result is contrary to a material-specific hypothesis (i.e., verbal and visual-spatial material are processed in the left and right hemispheres respectively) [95,111] as all task stimuli were displayed using Arabic numerals. Also, it is contrary to a rote memory vs procedure-based math problem-solving hypothesis (i.e., easy math is handled by rote memory in the left hemisphere) [79]. We suggest that this result may support a right-left-right hemispheric hypothesis that proposes that hemispheric implication is driven by a trade-off between familiarity and novelty, and a trade-off between the demand of the task (i.e., difficulty) and mental attentional capacity of the problem solver [60,97,112]. Finally, the associations between PMT scores and MdLF microstructural metrics are asymmetric with the relations with accuracy localized in the right hemisphere and being significant only for easy (one- and two-digit) tasks, and with reaction time – in the left hemisphere and for complex (three-digit) problems, mainly division. Importantly, age plays a critical role in mediating these associations, with the effects of diffusivity metrics on PMT scores diminishing with age. This suggests that although maturational aspects that contribute to microstructure and math abilities contribute to these relations earlier in life, other factors may contribute to these relations in adulthood. Results are discussed considering past empirical and theoretical reports.

### Math scores and age

Behavioural analyses showed significant correlations between PMT reaction time and age, as well as differences in reaction time scores among groups, primarily for difficulty levels 2 and 3. As the task complexity increased from one- to three-digit, reaction time slowed and accuracy dropped in all three age groups, which is consistent with previous studies [26,89,113]. Children performed significantly slower on most tasks whereas accuracy was mainly similar among groups, with some exceptions. On the one hand, this points to an age-related increase in processing speed, i.e., the speed of manipulating numeric information, including numeric fact retrieval, irrespective of the task, and, on the other hand, may indicate that within each math domain, performance or ability to solve arithmetic problems relies more on the overall complexity of the task. Arguably, harder tasks would require more time to solve and more resources for sequencing processing during problem solving. Considering that our paradigm is self-paced, from PMT performance scores we observe that participants generally slow down to attain accuracy when solving harder tasks, therefore most effects were observed in terms of reaction time. Alternatively, fewer associations with accuracy might indicate utilization of different cognitive strategies resulting in more pronounced individual variations in observed associations. Reaction time in cognitive tasks is known to be linked to the integrity of white matter fibre tracts across development [114,115] and has been documented for many major fibre tracts, such as the corpus callosum [116], right optic radiation [117], and right uncinate fasciculus [118]. Critically, developmental DTI data on MdLF are limited.

### MdLF DTI metrics and age

The relations among MdLF microstructural metrics and age are consistent with past research on other white matter fibre tracts [30,107–110,119,120]. Specifically, a lack or only slight changes in AD with age have been reported in a comprehensive study by Lebel and colleagues [121]. A tendency of FA to increase and RD and MD to decrease with age is evident from both between-group comparisons and correlation analyses. An increase in FA and a reduction of RD and MD values, generally exponential and often non-linear, from childhood to adulthood have been previously documented for the majority of association fibre tracts [122], including the temporal, frontal, and parietal white matter [123], with AD showing a more complex developmental profile [110,122,124,125]. These developmental changes may point to greater myelination, fibre density, spatial organization, and axonal elongation or reduction of axonal diameter and volume [50,110,126]. Given the proximity of the MdLF to these association fibre tracts, we may expect concurrent developmental changes in the MdLF microstructure. The correlations between age and MdLF microstructure being significant in the left hemisphere for FA and in the right hemisphere for RD might suggest asynchronous tract maturation, branching, or an increase in the number of fibres that run through the temporoparietal areas intersecting with the MdLF, such as the SLF and arcuate fasciculus (AF). These tracts exhibit larger volumes and therefore more extensive growth and myelination in the left hemisphere, potentially contributing to denser, more compact, and spatially organized white matter and increasing anisotropy in the MdLF [109,127]. Conversely, the lack of significant associations between FA in the right MdLF and age could indicate more uniform or earlier maturation within this portion of the tract, as FA can remain constant during neurodevelopment if the bundles mature at the same rate [49]. As there is no period in development when brain white matter microstructure is static [30,36,122], these DTI metric changes were expected, albeit only some reveal significant linear relations.

### Interhemispheric asymmetry of MdLF

In our study, the analysis of the whole-tract DTI metrics showed no interhemispheric differences in FA and a rightward asymmetry in AD, RD, and MD in all three groups. In particular, we document for the first time that AD, RD, and MD values in the right MdLF are significantly lower than in the left MdLF. As lower values of AD, RD, and MD correspond to advanced maturation in childhood, these findings suggest that the right hemisphere matures before the left hemisphere. This is consistent with the idea that the two hemispheres have cyclical developmental patterns as documented by electroencephalography [128,129] and ultrasonography metrics [130]. These alternating patterns have been suggested to reflect developmental stages [60,131].

Consideration of past results on the bilateral organization of the MdLF is inconclusive. For instance, Palejwala et al. [132] found no significant differences in tract volumes between the MdLF in the left and right hemispheres in adult participants. In contrast, de Champfleur and colleagues [133] reported a leftward asymmetry (i.e., the left hemisphere having higher FA values and thus being more mature) for the mean FA in the MdLF. In other work, Makris et al. [134] did not find significant left-right differences in the whole-tract metrics (FA, AD, and RD), but reported higher mean volume and FA in the right MdLF compared to the left MdLF, which did not reach a statistical significance level. They also revealed lateralization of MdLF connections with the left MdLF being predominantly comprised of fibres connecting the superior temporal cortex, i.e., dorsal temporal pole and superior temporal gyrus, and the inferior parietal lobule, and the right MdLF having more fibres connecting the superior temporal areas with the superior parietal lobule [134]. No differences in the whole-tract volume, length, or diffusivity metrics between left and right MdLF were detected by Latini et al. [24], although a small hemispheric asymmetry in connectivity pattern was found in the anterior portion of the MdLF with the left MdLF having more connections between the planum polare and inferior lateral occipital cortex. Makris and colleagues [134] highlighted high variability and various patterns of cortico-cortical connections within the MdLF which differed between the two hemispheres. In the left hemisphere, the most frequently observed connectivity pattern was the pathway between the temporal pole, superior temporal gyrus, and angular gyrus, and in the right hemisphere, the most common were the connections between the temporal pole, superior temporal gyrus, and superior parietal lobule.

Our findings show that some DTI metrics differ as a function of hemisphere (i.e., AD, RD, and MD), and some change with age (i.e., FA and RD), raising considerations on the biological nature and interpretability of the commonly used diffusion indices. A robust (*r *> 0.8) correlation between RD and MD in both hemispheres suggests that these two metrics may show similar trends in their relations with cognitive abilities. Interestingly, AD, often interpreted alongside MD and RD, also correlates positively with FA. Since higher FA values are known to reflect higher fibre density and the presence of a dominant fiber direction, opposite to RD and MD [36,49,135], this positive correlation complicates the straightforward interpretation of the relations between AD, age, and cognitive abilities, but suggests that AD may be viewed as an integral measure of anisotropy and diffusivity. In clinical samples, AD is thought to be indicative of axonal damage, including fragmentation of axons, disturbed microtubule arrangement, aggregation of filaments, and axonal transport, and in a healthy population, changes in axonal volumetric properties, particularly as a result of axonal growth and elongation [124,136–138]. Some support for this is evident from the animal studies showing the lack of changes in the values of AD along with increasing RD in dysmyelinated mice [126,138], a finding not yet documented in humans. MD is an informative and robust measure of underlying white matter microstructure, i.e., overall membrane integrity and cellular size. As it represents overall diffusion equally weighted along all three axes, MD may be sensitive to changes in AD and RD, or their proportion. RD, in turn, is more likely to be the best proxy for axonal diameter and, indirectly, (de)myelination, as it reflects the extent to which transverse diffusion is restricted [42,49]. FA is a relative and integral measure that reflects fibre spatial organization and directionality [108,139,140]. As it was explained in the elegant paper of Figley et al. [49], in the regions containing crossing fibres, FA reflects the direction of the dominant fibres but not the fibre amount or density itself. Further, the lack of differences in FA does not mean equivalent white matter structure, as FA may remain unchanged in the case of a proportional change in AD, RD, and MD, or the three eigenvalues that define FA.

Although currently there is no single “golden standard” DTI measure that can unambiguously determine variations in white matter microstructure across development, our results suggest that MdLF FA and RD may be better indicators of linear age effects. Also, right < left hemisphere values from diffusivity measures may indicate asynchronous myelination that can characterize typical development in children, adolescents, and adults. Overall, our results document that normal development patterns in DTI metrics in the MdLF can be useful in clinical practice and serve as markers for comparisons with children diagnosed with neurodevelopmental conditions.

### MdLF and math abilities: Effects of age and hemispheres

DTI metrics in bilateral MdLF significantly relate to mathematical scores which seem to cluster more based on difficulty and hemisphere. Specifically, associations between DTI metrics and math performance demonstrated hemispheric effects that favoured the right hemisphere for easier levels (one- and two-digit) and the left hemisphere for harder tasks (three-digit). Thus, both the right and left hemispheres share variance in their relation between DTI and math ability. Beyond age, shared variance in these associations may be driven by anything from genetics, environment (e.g., nutrition), or cognitive strategies employed during problem solving [141–144].

Critically, we emphasize that both hemispheres are involved in math problem solving. However, hemispheric asymmetry in our data is not consistent with the rote memory retrieval hypothesis (i.e., the left hemisphere handles math problems whose solutions are retrieved from memory [96]) or a material-specific hypothesis (i.e., verbal and spatial material are processed in the left and right hemispheres respectively [95]). Historically, initial findings by Roger Sperry in 1974 [111] on brain hemispheric specialization in mathematical processing suggested left hemisphere dominance, particularly in language-dependent arithmetic tasks [145], reflecting the knowledge of that era. Our results are more consistent with the right-left-right hemispheric hypothesis that proposes a dynamic hemispheric implication driven by a trade-off between familiarity and novelty, and a trade-off between the demand of the task (i.e., difficulty) and the mental attentional capacity of the problem solver [146]. Specifically, from a constructivist-developmental perspective [131,147], the right-left-right hypothesis suggests that the left hemisphere is primarily involved in analytical and effortful mental-attentional processing, often required in more challenging problem-solving scenarios that are within the problem-solvers ability. In contrast, the right hemisphere is associated with overlearned or automatized processing, engaged during either very easy or very difficult tasks. This hypothesis posits a trade-off between the mental demand of the task and the mental-attentional capacity of the individual to predict hemispheric dominance. This distinction is evidenced in tasks such as number processing, where easy tasks activate the right parietal cortex, while more complex calculation tasks engage the left parietal and frontal cortices [60].

Regression results highlight associations between DTI metrics and PMT performance mainly on division tasks. Some associations we observe agree with the literature, whereas others are rather unexpected. Specifically, we see lower FA predicting better accuracy in easy divisions tasks and higher RD and MD predicting faster reaction time in difficult division tasks. Lower FA may reflect either less complex circuitry, where streamlined pathways suffice for routine math problem solving as a result of skill mastery. Alternatively, it may indicate greater axonal branching and a more distributed connectivity supporting the recruitment of diffuse networks to aid parallel processing for basic computations [49]. This assumption agrees with the unexpected negative association between RD/MD and PMT reaction time. AD also showed a speed-accuracy trade-off: higher AD predicted faster reactions but lower accuracy. An expected finding is an inverse association between RD/MD and accuracy in easy division tasks, meaning that higher myelination and overall fibre integrity predict better performance, although this effect was modulated by age [148]. Thus, we speculate that some tasks may require fewer specialized resources, favouring widespread integration over rigid organization. While coherent axonal organization may accelerate signal propagation, benefiting reaction time, overly rigid pathways might hinder error monitoring, flexibility for error correction, alternative strategy recruitment, or iterative verification processes.

Although some findings are unexpected, we emphasize that this is the first study that looks at division tasks of three difficulty levels in the context of white matter microstructure. Division is unique among the four basic arithmetic operations due to its reliance on a combination of procedural strategies and reasoning skills, rather than primarily on fact retrieval from long-term memory [149]. Additionally, the learning sequence, where division is typically taught last, contributes to less proficiency in acquiring division facts, making division less conducive to direct retrieval strategies and necessitating laborious backup strategies for problem-solving [150]. Finally, cognitive strategies employed in solving division problems can create negative relations as other neighbouring fibre tracts take over. As this arithmetic operation is least studied, particularly with neuroimaging [61], further research is needed to understand brain correlates in division.

## Limitations

We recognize that there are key considerations associated with MRI/DTI and our study in particular. Specifically, head motion during MRI data collection remains the most prevalent artifact, especially in pediatric samples. To control for head motions we applied motion correction as a part of QIT’s pipeline. Participants with poor tract quality were excluded from the analysis. DTI provides a non-direct measure of white matter fibre orientation and microstructure, and the precision with which the fibre tracts are reconstructed heavily depends on the acquisition sequence and the type of analysis. Also, DTI relies on a Gaussian tensor model to represent water diffusion in brain tissue assuming a single, dominant fibre orientation within each voxel [151], which is often inadequate in complex white matter architecture. This simplification of the underlying anatomy becomes problematic in regions with complex fibre architectures, such as crossing, kissing, or fanning fibres. In regions of dense fibre crossings, due to partial volume effects the diffusion signal may reflect contributions from multiple tracts, reducing tracking precision (e.g., through false continuations or premature termination of streamlines). Specifically, the MdLF is anatomically close to other association tracts, including the ILF, IFOF, and the posterior segment of the AF [22–25], and due to this spatial overlap, traditional tractography algorithms may fail to reliably distinguish MdLF fibres from adjacent pathways, especially where tracts cross or run in parallel. Considering also variability in tractography methodologies which remains a challenge for cross-study comparisons, in our analyses, we used all four major diffusion metrics that characterize different aspects of white matter microstructure. We also recommend future research to consider other non-invasive methods for assessing white matter microstructure such as myelin water imaging [31,32,152,153], magnetization transfer imaging [154–156], *g*-ratio [34,157], and neurite orientation dispersion and density imaging [158,159], as well as constrained spherical deconvolution [37,160] that enables multi-directional streamline tracking.

Notably, the analyses we performed address questions about linear relations among variables, whereas structure-function relations may follow non-linear patterns. Further, specific to our study, albeit our samples were balanced in terms of sex, we did not have enough power to examine effects of sex. Finally, we used a cross-sectional design which has disadvantages compared to a longitudinal study. Although cross-sectional studies are more practical and affordable, future research should consider adopting a longitudinal approach as it is more suitable for capturing the dynamic changes that occur over time.

## Conclusions

We examine microstructural indices in bilateral MdLF and relate them to performance on arithmetic operations with three levels of difficulty in children, adolescents, and adults. We report for the first time diffusivity measures in the right MdLF are significantly lower than in the left MdLF, and that FA and RD may be better markers for linear age effects. Further, we document relations of DTI metrics with mathematical skills, showing hemispheric asymmetry. Linear associations between MdLF values with PMT scores favour easier tasks in the right hemisphere, and harder tasks in the left hemisphere. This is contrary to the classic hypothesis of hemisphere dominance but seems to adhere to the right-left-right hemispheric dominance hypothesis of a cognitive development theory of constructive operators [60,97,147]. We note also, that the role of age in mediating linear relations between MdLF microstructure and math performance differed among DTI metrics. Because relations among some variables are independent of age, further research is needed to decipher contributing factors which could be anything from strategy choice to genetics.

Overall, our results provide new insights into the structure of the MdLF across development and its relation to math problem solving and task difficulty. Importantly they highlight the role of the bilateral MdLF in mathematical tasks and contribute to theoretical understanding of the dynamic role of the hemispheres. These findings have practical and clinical importance as future studies can build on factors that contribute to brain-behaviour relations and their effects on neurodevelopmental disorders.

## Supporting information

S1 File **S1 Table.** PMT performance scores (accuracy and reaction time) as a function of age group and across three levels of difficulty for addition, subtraction, multiplication, and division tasks. **S2 Table.** DTI metrics (FA, AD, RD, and MD) in bilateral MdLF, as a function of age group. **S3 Table.** Pearson r and p-values for the correlations among DTI metrics (FA, AD, RD, and MD) in bilateral MdLF. **S4 Table.** Descriptive statistics (mean, SD) for PMT accuracy as a function of age group and across three levels of difficulty for addition, subtraction, multiplication, and division tasks. **S5 Table.** Descriptive statistics (mean, SD) for PMT reaction time as a function of age group and across three levels of difficulty for addition, subtraction, multiplication, and division tasks. **S6 Table.** Results of ANOVA analyses for PMT accuracy across three levels of difficulty for addition, subtraction, multiplication, and division tasks in children, adolescents, and adults. **S7 Table.** Results of ANOVA analyses for PMT reaction time across three levels of difficulty for addition, subtraction, multiplication, and division tasks in children, adolescents, and adults. **S8 Table.** Descriptive statistics (mean, SD) for DTI metrics (FA, AD, RD, and MD) in children, adolescents, and adults. **S9 Table.** Results of ANOVA analyses for DTI metrics (FA, AD, RD, and MD) in children, adolescents, and adults. **S10 Table.** Table Results of between-group comparison (t-test) for DTI metrics in the left and right MdLF. **S11 Table.** Results of linear regression models for each PMT performance score (accuracy, reaction time) and DTI metric (FA, AD, RD, and MD). **S12 Table.** Results of linear regression models for each PMT performance score (accuracy, reaction time) and DTI metric (FA, AD, RD, and MD) – model statistics. **S13 Table.** Pearson *r* and *p*-values for the correlations between PMT accuracy and DTI metric (FA, AD, RD, and MD) before and after controlling for age. **S14 Table.** Pearson *r* and *p*-values for the correlations between PMT reaction time and DTI metric (FA, AD, RD, and MD) before and after controlling for age.
(ZIP)

S1 Checklist XXX. (DOCX)
