# Supplementary material for: Math skills and microstructure of the middle longitudinal fasciculus: A developmental investigation
Source: PLoS One. 2025 Jun 11;20(6):e0324802. doi: 10.1371/journal.pone.0324802 (PMC12157344; doi:10.1371/journal.pone.0324802)
Supplement: S1 File — S1 Table. PMT performance scores (accuracy and reaction time) as a function of age group and across three levels of difficulty for addition, subtraction, multiplication, and division tasks. S2 Table. DTI metrics (FA, AD, RD, and MD) in bilateral MdLF, as a function of age group. S3 Table. Pearson r and p-values for the correlations among DTI metrics (FA, AD, RD, and MD) in bilateral MdLF. S4 Table. Descriptive statistics (mean, SD) for PMT accuracy as a function of age group and across three levels of difficulty for addition, subtraction, multiplication, and division tasks. S5 Table. Descriptive statistics (mean, SD) for PMT reaction time as a function of age group and across three levels of difficulty for addition, subtraction, multiplication, and division tasks. S6 Table. Results of ANOVA analyses for PMT accuracy across three levels of difficulty for addition, subtraction, multiplication, and division tasks in children, adolescents, and adults. S7 Table. Results of ANOVA analyses for PMT reaction time across three levels of difficulty for addition, subtraction, multiplication, and division tasks in children, adolescents, and adults. S8 Table. Descriptive statistics (mean, SD) for DTI metrics (FA, AD, RD, and MD) in children, adolescents, and adults. S9 Table. Results of ANOVA analyses for DTI metrics (FA, AD, RD, and MD) in children, adolescents, and adults. S10 Table. Table Results of between-group comparison (t-test) for DTI metrics in the left and right MdLF. S11 Table. Results of linear regression models for each PMT performance score (accuracy, reaction time) and DTI metric (FA, AD, RD, and MD). S12 Table. Results of linear regression models for each PMT performance score (accuracy, reaction time) and DTI metric (FA, AD, RD, and MD) – model statistics. S13 Table. Pearson r and p-values for the correlations between PMT accuracy and DTI metric (FA, AD, RD, and MD) before and after controlling for age. S14 Table. Pearson r and p-values for the correlations betw [file pone.0324802.s001.zip › Supporting information_update210525/S12 Table.Results of linear regression models for each PMT performance score (accuracy, reaction time) and DTI metric (FA, AD, RD, and MD) ΓÇô model statistics.pdf]

S12 Table. Results of linear regression models for each PMT performance score (accuracy, reaction time) and DTI metric (FA, AD, RD, and MD) – model statistics

Model	$R2$	Adjusted $R2$	F	$F(p)$
ACC ADD1~FA	0,191	0,111	2,367	0,053
ACC ADD2~FA	0,118	0,030	1,344	0,262
ACC ADD3~FA	0,149	0,063	1,745	0,142
ACC SUB1~FA	0,105	0,016	1,175	0,335
ACC SUB2~FA	0,123	0,035	1,397	0,242
ACC SUB3~FA	0,117	0,029	1,330	0,267
ACC MUL1~FA	0,108	0,018	1,205	0,320
ACC MUL2~FA	0,114	0,025	1,285	0,285
ACC MUL3~FA	0,261	0,187	3,524	**0,008**
ACC DIV1~FA	0,175	0,093	2,127	0,077
ACC DIV2~FA	0,089	-0,002	0,977	0,441
ACC DIV3~FA	0,063	-0,031	0,671	0,647
RT ADD1~FA	0,314	0,245	4,575	**0,002**
RT ADD2~FA	0,294	0,224	4,174	**0,003**
RT ADD3~FA	0,208	0,129	2,624	**0,035**
RT SUB1~FA	0,185	0,104	2,270	0,062
RT SUB2~FA	0,101	0,011	1,126	0,359
RT SUB3~FA	0,217	0,139	2,775	**0,027**
RT MUL1~FA	0,295	0,224	4,180	**0,003**
RT MUL2~FA	0,299	0,229	4,265	**0,003**
RT MUL3~FA	0,273	0,200	3,751	**0,006**
RT DIV1~FA	0,225	0,147	2,901	**0,022**
RT DIV2~FA	0,214	0,136	2,729	**0,030**
RT DIV3~FA	0,063	-0,030	0,675	0,645
ACC ADD1~AD	0,167	0,083	2,001	0,095
ACC ADD2~AD	0,094	0,003	1,036	0,407

ACC ADD3~AD	0,172	0,090	2,082	0,083
ACC SUB1~AD	0,142	0,057	1,659	0,162
ACC SUB2~AD	0,231	0,154	3,000	**0,019**
ACC SUB3~AD	0,064	-0,029	0,687	0,636
ACC MUL1~AD	0,063	-0,030	0,675	0,644
ACC MUL2~AD	0,202	0,122	2,534	**0,040**
ACC MUL3~AD	0,197	0,117	2,457	**0,046**
ACC DIV1~AD	0,180	0,098	2,190	0,070
ACC DIV2~AD	0,214	0,136	2,729	**0,030**
ACC DIV3~AD	0,074	-0,019	0,795	0,558
RT ADD1~AD	0,318	0,250	4,662	**0,001**
RT ADD2~AD	0,251	0,177	3,358	**0,011**
RT ADD3~AD	0,126	0,039	1,443	0,225
RT SUB1~AD	0,162	0,079	1,939	0,104
RT SUB2~AD	0,089	-0,003	0,972	0,444
RT SUB3~AD	0,210	0,131	2,658	**0,033**
RT MUL1~AD	0,323	0,255	4,765	**0,001**
RT MUL2~AD	0,231	0,154	3,008	**0,019**
RT MUL3~AD	0,237	0,160	3,101	**0,016**
RT DIV1~AD	0,178	0,096	2,169	0,072
RT DIV2~AD	0,140	0,054	1,627	0,170
RT DIV3~AD	0,119	0,031	1,350	0,259
ACC ADD1~RD	0,196	0,116	2,444	**0,047**
ACC ADD2~RD	0,087	-0,004	0,951	0,456
ACC ADD3~RD	0,071	-0,022	0,762	0,582
ACC SUB1~RD	0,008	-0,091	0,079	0,995
ACC SUB2~RD	0,048	-0,048	0,499	0,776
ACC SUB3~RD	0,040	-0,056	0,415	0,836
ACC MUL1~RD	0,092	0,001	1,008	0,423
ACC MUL2~RD	0,115	0,027	1,304	0,277

ACC MUL3~RD	0,264	0,190	3,587	**0,008**
ACC DIV1~RD	0,049	-0,046	0,514	0,764
ACC DIV2~RD	0,134	0,048	1,551	0,191
ACC DIV3~RD	0,078	-0,015	0,842	0,526
RT ADD1~RD	0,290	0,219	4,092	**0,003**
RT ADD2~RD	0,228	0,151	2,955	**0,021**
RT ADD3~RD	0,188	0,107	2,312	0,058
RT SUB1~RD	0,103	0,013	1,143	0,351
RT SUB2~RD	0,103	0,014	1,154	0,345
RT SUB3~RD	0,162	0,079	1,938	0,105
RT MUL1~RD	0,270	0,197	3,706	**0,006**
RT MUL2~RD	0,220	0,142	2,827	**0,025**
RT MUL3~RD	0,232	0,155	3,024	**0,018**
RT DIV1~RD	0,120	0,032	1,361	0,255
RT DIV2~RD	0,171	0,088	2,056	0,087
RT DIV3~RD	0,182	0,100	2,224	0,066
ACC ADD1~MD	0,190	0,108	2,338	0,055
ACC ADD2~MD	0,108	0,019	1,214	0,316
ACC ADD3~MD	0,123	0,036	1,406	0,238
ACC SUB1~MD	0,060	-0,034	0,642	0,669
ACC SUB2~MD	0,155	0,071	1,839	0,122
ACC SUB3~MD	0,049	-0,046	0,518	0,762
ACC MUL1~MD	0,068	-0,025	0,730	0,604
ACC MUL2~MD	0,186	0,105	2,292	0,059
ACC MUL3~MD	0,230	0,153	2,985	**0,020**
ACC DIV1~MD	0,101	0,011	1,121	0,362
ACC DIV2~MD	0,208	0,129	2,634	**0,034**
ACC DIV3~MD	0,083	-0,009	0,900	0,489
RT ADD1~MD	0,301	0,231	4,308	**0,002**
RT ADD2~MD	0,219	0,141	2,807	**0,026**

RT ADD3~MD	0,156	0,072	1,850	0,120
RT SUB1~MD	0,109	0,020	1,220	0,313
RT SUB2~MD	0,090	-0,001	0,987	0,435
RT SUB3~MD	0,184	0,103	2,257	0,063
RT MUL1~MD	0,303	0,233	4,346	**0,002**
RT MUL2~MD	0,198	0,118	2,465	**0,045**
RT MUL3~MD	0,237	0,161	3,115	**0,016**
RT DIV1~MD	0,130	0,043	1,494	0,208
RT DIV2~MD	0,145	0,059	1,695	0,153
RT DIV3~MD	0,187	0,106	2,307	0,058

ACC – accuracy, RT – reaction time, ADD – addition, SUB – subtraction, MUL – multiplication, DIV – division. FA – fractional anisotropy, AD – axial diffusivity, RD – radial diffusivity, MD – mean diffusivity, MdLF – middle longitudinal fasciculus. 1, 2, and 3 correspond to difficulty levels 1, 2, and 3, respectively. Model: PMT score ~ age(DTI left MdLF + DTI right MdLF).*
