# Supplementary material for: Math skills and microstructure of the middle longitudinal fasciculus: A developmental investigation
Source: PLoS One. 2025 Jun 11;20(6):e0324802. doi: 10.1371/journal.pone.0324802 (PMC12157344; doi:10.1371/journal.pone.0324802)
Supplement: S1 File — S1 Table. PMT performance scores (accuracy and reaction time) as a function of age group and across three levels of difficulty for addition, subtraction, multiplication, and division tasks. S2 Table. DTI metrics (FA, AD, RD, and MD) in bilateral MdLF, as a function of age group. S3 Table. Pearson r and p-values for the correlations among DTI metrics (FA, AD, RD, and MD) in bilateral MdLF. S4 Table. Descriptive statistics (mean, SD) for PMT accuracy as a function of age group and across three levels of difficulty for addition, subtraction, multiplication, and division tasks. S5 Table. Descriptive statistics (mean, SD) for PMT reaction time as a function of age group and across three levels of difficulty for addition, subtraction, multiplication, and division tasks. S6 Table. Results of ANOVA analyses for PMT accuracy across three levels of difficulty for addition, subtraction, multiplication, and division tasks in children, adolescents, and adults. S7 Table. Results of ANOVA analyses for PMT reaction time across three levels of difficulty for addition, subtraction, multiplication, and division tasks in children, adolescents, and adults. S8 Table. Descriptive statistics (mean, SD) for DTI metrics (FA, AD, RD, and MD) in children, adolescents, and adults. S9 Table. Results of ANOVA analyses for DTI metrics (FA, AD, RD, and MD) in children, adolescents, and adults. S10 Table. Table Results of between-group comparison (t-test) for DTI metrics in the left and right MdLF. S11 Table. Results of linear regression models for each PMT performance score (accuracy, reaction time) and DTI metric (FA, AD, RD, and MD). S12 Table. Results of linear regression models for each PMT performance score (accuracy, reaction time) and DTI metric (FA, AD, RD, and MD) – model statistics. S13 Table. Pearson r and p-values for the correlations between PMT accuracy and DTI metric (FA, AD, RD, and MD) before and after controlling for age. S14 Table. Pearson r and p-values for the correlations betw [file pone.0324802.s001.zip › Supporting information_update210525/S6 Table.Results of ANOVA analyses for PMT accuracy across three levels of addition, subtraction, multiplication, and division tasks in children, adolescents, and adults.pdf]

S6 Table. Results of ANOVA analyses for PMT accuracy across three levels of addition, subtraction, multiplication, and division tasks in children, adolescents, and adults

Addition Level 1

	Df	**Sum of Squares**	**Mean Square**	***F***	***F*(*p*)**	**Mean Difference**	**CI Lower Bound**	**CI Upper Bound**	***p*-value**	**Adjusted *p*-value**
**Age group**	2	0,0167	0,0083	4,84	0,012	2-1	0,029	-0,002	0,060	0,068
**Residuals**	53	0,0912	0,0017			3-1	0,041	0,007	0,075	**0,015**
						3-2	0,012	-0,023	0,047	0,692

Addition Level 2

	Df	**Sum of Squares**	**Mean Square**	***F***	***F*(*p*)**	
**Age group**	2	0,0461	0,0230	1,763	0,181	No Tukey's HSD test performed
**Residuals**	53	0,6924	0,0131			

Addition Level 3

	Df	**Sum of Squares**	**Mean Square**	***F***	***F*(*p*)**	
**Age group**	2	0,1202	0,0601	1,447	0,244	No Tukey's HSD test performed
**Residuals**	53	2,2021	0,0416			

Subtraction Level 1

	Df	**Sum of Squares**	**Mean Square**	***F***	***F*(*p*)**	
**Age group**	2	0,0014	0,0007	0,35	0,706	No Tukey's HSD test performed
**Residuals**	53	0,1073	0,0020			

Subtraction Level 2

	Df	**Sum of Squares**	**Mean Square**	***F***	***F*(*p*)**	
**Age group**	2	0,0109	0,0054	0,293	0,747	No Tukey's HSD test performed
**Residuals**	53	0,9827	0,0185			

Subtraction Level 3

	Df	**Sum of Squares**	**Mean Square**	***F***	***F*(*p*)**	
**Age group**	2	0,0600	0,0302	0,425	0,656	No Tukey's HSD test performed
**Residuals**	53	3,7750	0,0712			

Multiplication Level 1

	Df	**Sum of Squares**	**Mean Square**	***F***	***F*(*p*)**	
**Age group**	2	0,0143	0,0071	3,076	0,055	No Tukey's HSD test performed
**Residuals**	53	0,1232	0,0023			

Multiplication Level 2

	Df	**Sum of Squares**	**Mean Square**	***F***	***F*(*p*)**	
**Age group**	2	0,1917	0,0959	2,66	0,079	No Tukey's HSD test performed
**Residuals**	53	1,9105	0,0361			

Multiplication Level 3

	Df	**Sum of Squares**	**Mean Square**	***F***	***F*(*p*)**	**Mean Difference**	**CI Lower Bound**	**CI Upper Bound**	***p*-value**	**Adjusted *p*-value**
**Age group**	2	1,1730	0,5866	7,712	0,001	2-1	0,223	0,017	0,428	**0,031**
**Residuals**	53	4,0320	0,0761			3-1	0,355	0,128	0,583	**0,001**
						3-2	0,133	-0,099	0,364	0,359

Division Level 1

	Df	**Sum of Squares**	**Mean Square**	***F***	***F*(*p*)**	
**Age group**	2	0,0204	0,0102	0,954	0,392	No Tukey's HSD test performed
**Residuals**	53	0,5655	0,0107			

Division Level 2

	Df	**Sum of Squares**	**Mean Square**	***F***	***F*(*p*)**	
**Age group**	2	0,1396	0,0698	1,188	0,313	No Tukey's HSD test performed
**Residuals**	53	3,1130	0,0587			

Division Level 3

	Df	**Sum of Squares**	**Mean Square**	***F***	***F*(*p*)**	
**Age group**	2	0,2250	0,1126	1,112	0,337	No Tukey's HSD test performed
**Residuals**	53	5,3700	0,1013			

CI – confidence interval, Df – degrees of freedom, F(p) – p-value for F-statistic. Tukey's HSD – Tukey's Honestly Significant Difference. Age groups 1, 2, and 3 correspond to children, adolescents, and adults, respectively. Tukey multiple comparisons of means was performed at 95% family-wise confidence level.
