# Supplementary material for: Math skills and microstructure of the middle longitudinal fasciculus: A developmental investigation
Source: PLoS One. 2025 Jun 11;20(6):e0324802. doi: 10.1371/journal.pone.0324802 (PMC12157344; doi:10.1371/journal.pone.0324802)
Supplement: S1 File — S1 Table. PMT performance scores (accuracy and reaction time) as a function of age group and across three levels of difficulty for addition, subtraction, multiplication, and division tasks. S2 Table. DTI metrics (FA, AD, RD, and MD) in bilateral MdLF, as a function of age group. S3 Table. Pearson r and p-values for the correlations among DTI metrics (FA, AD, RD, and MD) in bilateral MdLF. S4 Table. Descriptive statistics (mean, SD) for PMT accuracy as a function of age group and across three levels of difficulty for addition, subtraction, multiplication, and division tasks. S5 Table. Descriptive statistics (mean, SD) for PMT reaction time as a function of age group and across three levels of difficulty for addition, subtraction, multiplication, and division tasks. S6 Table. Results of ANOVA analyses for PMT accuracy across three levels of difficulty for addition, subtraction, multiplication, and division tasks in children, adolescents, and adults. S7 Table. Results of ANOVA analyses for PMT reaction time across three levels of difficulty for addition, subtraction, multiplication, and division tasks in children, adolescents, and adults. S8 Table. Descriptive statistics (mean, SD) for DTI metrics (FA, AD, RD, and MD) in children, adolescents, and adults. S9 Table. Results of ANOVA analyses for DTI metrics (FA, AD, RD, and MD) in children, adolescents, and adults. S10 Table. Table Results of between-group comparison (t-test) for DTI metrics in the left and right MdLF. S11 Table. Results of linear regression models for each PMT performance score (accuracy, reaction time) and DTI metric (FA, AD, RD, and MD). S12 Table. Results of linear regression models for each PMT performance score (accuracy, reaction time) and DTI metric (FA, AD, RD, and MD) – model statistics. S13 Table. Pearson r and p-values for the correlations between PMT accuracy and DTI metric (FA, AD, RD, and MD) before and after controlling for age. S14 Table. Pearson r and p-values for the correlations betw [file pone.0324802.s001.zip › Supporting information_update210525/S11 Table.Results of linear regression models for each PMT performance score (accuracy, reaction time) and DTI metric (FA, AD, RD, and MD).pdf]

S11 Table. Results of linear regression models for each PMT performance score (accuracy, reaction time) and DTI metric (FA, AD, RD, and MD)

Model	Variables	Estimate	Standardized Estimate	Standard Error	*t*	*p*
ACC ADD1~FA	Intercept	0,891	NA	0,019	45,879	0,000
ACC ADD1~FA	age	0,003	0,341	0,001	2,583	0,013
ACC ADD1~FA	FA left	-0,045	-1,015	0,024	-1,845	0,071
ACC ADD1~FA	FA right	-0,001	-0,030	0,026	-0,052	0,959
ACC ADD1~FA	age:FA left	0,003	1,079	0,002	1,957	0,056
ACC ADD1~FA	age:FA right	0,000	-0,115	0,002	-0,20	0,845
ACC ADD2~FA	Intercept	0,786	NA	0,053	14,815	0,000
ACC ADD2~FA	age	0,004	0,194	0,003	1,403	0,167
ACC ADD2~FA	FA left	-0,103	-0,893	0,067	-1,554	0,126
ACC ADD2~FA	FA right	-0,041	-0,358	0,070	-0,594	0,555
ACC ADD2~FA	age:FA left	0,007	0,936	0,004	1,625	0,110
ACC ADD2~FA	age:FA right	0,002	0,231	0,004	0,38	0,705
ACC ADD3~FA	Intercept	0,640	NA	0,092	6,919	0,000
ACC ADD3~FA	age	0,007	0,166	0,005	1,223	0,227
ACC ADD3~FA	FA left	0,119	0,580	0,116	1,027	0,309
ACC ADD3~FA	FA right	-0,180	-0,877	0,122	-1,482	0,145
ACC ADD3~FA	age:FA left	-0,003	-0,263	0,007	-0,465	0,644
ACC ADD3~FA	age:FA right	0,007	0,572	0,008	0,96	0,343
ACC SUB1~FA	Intercept	0,939	NA	0,021	45,800	0,000
ACC SUB1~FA	age	0,001	0,077	0,001	0,556	0,581
ACC SUB1~FA	FA left	0,008	0,169	0,026	0,293	0,771
ACC SUB1~FA	FA right	-0,044	-0,992	0,027	-1,634	0,108
ACC SUB1~FA	age:FA left	-0,001	-0,232	0,002	-0,400	0,691
ACC SUB1~FA	age:FA right	0,002	0,780	0,002	1,27	0,208

ACC SUB2~FA	Intercept	0,804	NA	0,061	13,094	0,000
ACC SUB2~FA	age	0,002	0,084	0,004	0,611	0,544
ACC SUB2~FA	FA left	0,008	0,061	0,077	0,106	0,916
ACC SUB2~FA	FA right	-0,145	-1,075	0,081	-1,789	0,080
ACC SUB2~FA	age:FA left	0,001	0,152	0,005	0,264	0,793
ACC SUB2~FA	age:FA right	0,006	0,775	0,005	1,28	0,207
ACC SUB3~FA	Intercept	0,693	NA	0,121	5,727	0,000
ACC SUB3~FA	age	-0,003	-0,053	0,007	-0,383	0,703
ACC SUB3~FA	FA left	-0,055	-0,206	0,152	-0,359	0,721
ACC SUB3~FA	FA right	-0,289	-1,096	0,159	-1,818	0,075
ACC SUB3~FA	age:FA left	0,007	0,428	0,009	0,742	0,461
ACC SUB3~FA	age:FA right	0,016	0,948	0,010	1,56	0,125
ACC MUL1~FA	Intercept	0,921	NA	0,023	39,976	0,000
ACC MUL1~FA	age	0,002	0,214	0,001	1,541	0,130
ACC MUL1~FA	FA left	0,014	0,284	0,029	0,491	0,626
ACC MUL1~FA	FA right	-0,003	-0,062	0,030	-0,102	0,919
ACC MUL1~FA	age:FA left	-0,001	-0,225	0,002	-0,388	0,700
ACC MUL1~FA	age:FA right	-0,001	-0,220	0,002	-0,36	0,720
ACC MUL2~FA	Intercept	0,604	NA	0,090	6,725	0,000
ACC MUL2~FA	age	0,011	0,284	0,005	2,050	0,046
ACC MUL2~FA	FA left	0,009	0,047	0,113	0,082	0,935
ACC MUL2~FA	FA right	-0,135	-0,689	0,118	-1,140	0,260
ACC MUL2~FA	age:FA left	0,000	-0,041	0,007	-0,071	0,943
ACC MUL2~FA	age:FA right	0,007	0,554	0,007	0,91	0,367
ACC MUL3~FA	Intercept	0,183	NA	0,129	1,421	0,162
ACC MUL3~FA	age	0,022	0,363	0,008	2,873	0,006
ACC MUL3~FA	FA left	0,107	0,346	0,162	0,658	0,513
ACC MUL3~FA	FA right	0,021	0,069	0,170	0,124	0,901

ACC MUL3~FA	age:FA left	0,000	0,009	0,010	0,018	0,986
ACC MUL3~FA	age:FA right	-0,007	-0,350	0,011	-0,63	0,532
ACC DIV1~FA	Intercept	0,847	NA	0,046	18,526	0,000
ACC DIV1~FA	age	0,004	0,209	0,003	1,567	0,123
ACC DIV1~FA	FA left	0,005	0,044	0,057	0,080	0,936
ACC DIV1~FA	FA right	-0,134	-1,303	0,060	-2,236	0,030
ACC DIV1~FA	age:FA left	-0,001	-0,080	0,004	-0,144	0,886
ACC DIV1~FA	age:FA right	0,007	1,075	0,004	1,83	0,073
ACC DIV2~FA	Intercept	0,636	NA	0,113	5,615	0,000
ACC DIV2~FA	age	0,009	0,185	0,007	1,320	0,193
ACC DIV2~FA	FA left	-0,105	-0,432	0,142	-0,740	0,463
ACC DIV2~FA	FA right	-0,097	-0,397	0,149	-0,648	0,520
ACC DIV2~FA	age:FA left	0,008	0,568	0,009	0,971	0,336
ACC DIV2~FA	age:FA right	0,003	0,216	0,009	0,35	0,727
ACC DIV3~FA	Intercept	0,397	NA	0,151	2,636	0,011
ACC DIV3~FA	age	0,012	0,189	0,009	1,331	0,189
ACC DIV3~FA	FA left	-0,102	-0,319	0,189	-0,539	0,592
ACC DIV3~FA	FA right	0,166	0,521	0,198	0,839	0,405
ACC DIV3~FA	age:FA left	0,008	0,418	0,012	0,705	0,484
ACC DIV3~FA	age:FA right	-0,012	-0,631	0,012	-1,01	0,319
RT ADD1~FA	Intercept	2,717	NA	0,251	10,846	0,000
RT ADD1~FA	age	-0,062	-0,512	0,015	-4,203	0,000
RT ADD1~FA	FA left	-0,088	-0,141	0,314	-0,279	0,782
RT ADD1~FA	FA right	0,235	0,379	0,330	0,712	0,480
RT ADD1~FA	age:FA left	-0,001	-0,021	0,019	-0,042	0,967
RT ADD1~FA	age:FA right	-0,007	-0,187	0,021	-0,35	0,728
RT ADD2~FA	Intercept	6,867	NA	0,723	9,495	0,000
RT ADD2~FA	age	-0,132	-0,381	0,043	-3,088	0,003

RT ADD2~FA	FA left	-1,313	-0,744	0,907	-1,448	0,154
RT ADD2~FA	FA right	0,650	0,368	0,952	0,683	0,498
RT ADD2~FA	age:FA left	0,043	0,400	0,056	0,777	0,441
RT ADD2~FA	age:FA right	-0,020	-0,181	0,060	-0,33	0,740
RT ADD3~FA	Intercept	10,205	NA	1,330	7,671	0,000
RT ADD3~FA	age	-0,140	-0,232	0,079	-1,773	0,082
RT ADD3~FA	FA left	-2,998	-0,978	1,669	-1,797	0,078
RT ADD3~FA	FA right	-0,357	-0,116	1,750	-0,204	0,839
RT ADD3~FA	age:FA left	0,129	0,684	0,103	1,254	0,216
RT ADD3~FA	age:FA right	0,035	0,184	0,110	0,32	0,751
RT SUB1~FA	Intercept	3,560	NA	0,633	5,621	0,000
RT SUB1~FA	age	-0,087	-0,307	0,038	-2,317	0,025
RT SUB1~FA	FA left	0,046	0,032	0,794	0,058	0,954
RT SUB1~FA	FA right	1,331	0,925	0,833	1,597	0,117
RT SUB1~FA	age:FA left	-0,010	-0,109	0,049	-0,197	0,845
RT SUB1~FA	age:FA right	-0,058	-0,653	0,052	-1,12	0,269
RT SUB2~FA	Intercept	7,959	NA	1,138	6,996	0,000
RT SUB2~FA	age	-0,117	-0,241	0,067	-1,729	0,090
RT SUB2~FA	FA left	-0,470	-0,191	1,427	-0,329	0,743
RT SUB2~FA	FA right	1,030	0,419	1,497	0,688	0,495
RT SUB2~FA	age:FA left	0,002	0,012	0,088	0,020	0,984
RT SUB2~FA	age:FA right	-0,041	-0,266	0,094	-0,43	0,666
RT SUB3~FA	Intercept	15,561	NA	2,507	6,206	0,000
RT SUB3~FA	age	-0,248	-0,217	0,149	-1,671	0,101
RT SUB3~FA	FA left	-5,602	-0,964	3,145	-1,781	0,081
RT SUB3~FA	FA right	-1,558	-0,268	3,299	-0,472	0,639
RT SUB3~FA	age:FA left	0,242	0,677	0,194	1,249	0,218
RT SUB3~FA	age:FA right	0,103	0,285	0,207	0,50	0,620

RT MUL1~FA	Intercept	3,782	NA	0,420	9,014	0,000
RT MUL1~FA	age	-0,099	-0,491	0,025	-3,982	0,000
RT MUL1~FA	FA left	-0,013	-0,012	0,526	-0,024	0,981
RT MUL1~FA	FA right	0,682	0,666	0,552	1,236	0,222
RT MUL1~FA	age:FA left	-0,006	-0,103	0,032	-0,199	0,843
RT MUL1~FA	age:FA right	-0,032	-0,496	0,035	-0,91	0,366
RT MUL2~FA	Intercept	11,158	NA	1,435	7,774	0,000
RT MUL2~FA	age	-0,241	-0,349	0,085	-2,838	0,007
RT MUL2~FA	FA left	-3,347	-0,952	1,800	-1,859	0,069
RT MUL2~FA	FA right	1,731	0,492	1,888	0,916	0,364
RT MUL2~FA	age:FA left	0,127	0,589	0,111	1,148	0,256
RT MUL2~FA	age:FA right	-0,070	-0,320	0,118	-0,59	0,557
RT MUL3~FA	Intercept	19,242	NA	2,579	7,460	0,000
RT MUL3~FA	age	-0,360	-0,295	0,153	-2,353	0,023
RT MUL3~FA	FA left	-5,857	-0,944	3,235	-1,810	0,076
RT MUL3~FA	FA right	-2,083	-0,336	3,394	-0,614	0,542
RT MUL3~FA	age:FA left	0,232	0,609	0,199	1,164	0,250
RT MUL3~FA	age:FA right	0,177	0,459	0,213	0,83	0,409
RT DIV1~FA	Intercept	4,484	NA	0,745	6,022	0,000
RT DIV1~FA	age	-0,123	-0,359	0,044	-2,777	0,008
RT DIV1~FA	FA left	0,889	0,513	0,934	0,952	0,346
RT DIV1~FA	FA right	1,341	0,773	0,980	1,368	0,177
RT DIV1~FA	age:FA left	-0,052	-0,492	0,058	-0,911	0,367
RT DIV1~FA	age:FA right	-0,058	-0,539	0,061	-0,95	0,348
RT DIV2~FA	Intercept	12,189	NA	1,881	6,480	0,000
RT DIV2~FA	age	-0,245	-0,287	0,111	-2,201	0,032
RT DIV2~FA	FA left	-2,337	-0,537	2,359	-0,990	0,327
RT DIV2~FA	FA right	-1,234	-0,284	2,475	-0,499	0,620

RT DIV2~FA	age:FA left	0,059	0,220	0,145	0,404	0,688
RT DIV2~FA	age:FA right	0,105	0,387	0,155	0,67	0,503
RT DIV3~FA	Intercept	15,481	NA	2,740	5,650	0,000
RT DIV3~FA	age	-0,186	-0,163	0,162	-1,148	0,257
RT DIV3~FA	FA left	4,381	0,755	3,437	1,275	0,208
RT DIV3~FA	FA right	-1,344	-0,231	3,605	-0,373	0,711
RT DIV3~FA	age:FA left	-0,231	-0,648	0,212	-1,092	0,280
RT DIV3~FA	age:FA right	0,106	0,295	0,226	0,47	0,640
ACC ADD1~AD	Intercept	0,901	NA	0,020	46,045	0,000
ACC ADD1~AD	age	0,003	0,292	0,001	2,154	0,036
ACC ADD1~AD	AD left	0,016	0,371	0,026	0,621	0,538
ACC ADD1~AD	AD right	0,002	0,049	0,025	0,087	0,931
ACC ADD1~AD	age:AD left	-0,001	-0,323	0,002	-0,506	0,615
ACC ADD1~AD	age:AD right	-0,001	-0,275	0,002	-0,45	0,653
ACC ADD2~AD	Intercept	0,805	NA	0,053	15,078	0,000
ACC ADD2~AD	age	0,004	0,175	0,003	1,240	0,221
ACC ADD2~AD	AD left	0,026	0,229	0,072	0,367	0,715
ACC ADD2~AD	AD right	-0,084	-0,723	0,068	-1,229	0,225
ACC ADD2~AD	age:AD left	-0,002	-0,241	0,005	-0,361	0,719
ACC ADD2~AD	age:AD right	0,004	0,543	0,005	0,86	0,396
ACC ADD3~AD	Intercept	0,623	NA	0,091	6,884	0,000
ACC ADD3~AD	age	0,008	0,201	0,005	1,485	0,144
ACC ADD3~AD	AD left	0,138	0,673	0,122	1,130	0,264
ACC ADD3~AD	AD right	-0,209	-1,018	0,116	-1,810	0,076
ACC ADD3~AD	age:AD left	-0,006	-0,477	0,008	-0,749	0,457
ACC ADD3~AD	age:AD right	0,009	0,680	0,008	1,12	0,268
ACC SUB1~AD	Intercept	0,948	NA	0,020	47,553	0,000
ACC SUB1~AD	age	0,000	0,026	0,001	0,188	0,851

ACC SUB1~AD	AD left	0,022	0,492	0,027	0,812	0,421
ACC SUB1~AD	AD right	-0,031	-0,707	0,025	-1,235	0,223
ACC SUB1~AD	age:AD left	-0,002	-0,575	0,002	-0,886	0,380
ACC SUB1~AD	age:AD right	0,001	0,421	0,002	0,68	0,499
ACC SUB2~AD	Intercept	0,829	NA	0,057	14,520	0,000
ACC SUB2~AD	age	0,001	0,042	0,003	0,325	0,747
ACC SUB2~AD	AD left	-0,020	-0,149	0,077	-0,260	0,796
ACC SUB2~AD	AD right	-0,116	-0,862	0,073	-1,591	0,118
ACC SUB2~AD	age:AD left	0,003	0,388	0,005	0,631	0,531
ACC SUB2~AD	age:AD right	0,003	0,337	0,005	0,58	0,567
ACC SUB3~AD	Intercept	0,709	NA	0,124	5,730	0,000
ACC SUB3~AD	age	-0,003	-0,048	0,007	-0,337	0,737
ACC SUB3~AD	AD left	0,080	0,303	0,167	0,478	0,634
ACC SUB3~AD	AD right	-0,127	-0,480	0,158	-0,802	0,426
ACC SUB3~AD	age:AD left	-0,001	-0,090	0,011	-0,132	0,895
ACC SUB3~AD	age:AD right	0,003	0,174	0,011	0,27	0,789
ACC MUL1~AD	Intercept	0,919	NA	0,023	39,222	0,000
ACC MUL1~AD	age	0,002	0,227	0,001	1,578	0,121
ACC MUL1~AD	AD left	0,017	0,335	0,032	0,528	0,600
ACC MUL1~AD	AD right	-0,027	-0,537	0,030	-0,898	0,373
ACC MUL1~AD	age:AD left	-0,001	-0,452	0,002	-0,667	0,508
ACC MUL1~AD	age:AD right	0,002	0,558	0,002	0,86	0,391
ACC MUL2~AD	Intercept	0,627	NA	0,085	7,412	0,000
ACC MUL2~AD	age	0,010	0,261	0,005	1,968	0,055
ACC MUL2~AD	AD left	0,076	0,388	0,114	0,664	0,510
ACC MUL2~AD	AD right	-0,198	-1,014	0,108	-1,837	0,072
ACC MUL2~AD	age:AD left	-0,005	-0,378	0,008	-0,604	0,548
ACC MUL2~AD	age:AD right	0,009	0,728	0,007	1,22	0,227

ACC MUL3~AD	Intercept	0,112	NA	0,133	0,841	0,404
ACC MUL3~AD	age	0,026	0,432	0,008	3,243	0,002
ACC MUL3~AD	AD left	0,113	0,369	0,181	0,628	0,533
ACC MUL3~AD	AD right	0,032	0,103	0,170	0,186	0,854
ACC MUL3~AD	age:AD left	-0,008	-0,414	0,012	-0,659	0,513
ACC MUL3~AD	age:AD right	-0,001	-0,066	0,012	-0,11	0,912
ACC DIV1~AD	Intercept	0,854	NA	0,045	18,881	0,000
ACC DIV1~AD	age	0,004	0,215	0,003	1,594	0,117
ACC DIV1~AD	AD left	0,087	0,841	0,061	1,418	0,163
ACC DIV1~AD	AD right	-0,159	-1,540	0,058	-2,752	0,008
ACC DIV1~AD	age:AD left	-0,006	-0,849	0,004	-1,338	0,187
ACC DIV1~AD	age:AD right	0,009	1,396	0,004	2,31	0,025
ACC DIV2~AD	Intercept	0,616	NA	0,104	5,899	0,000
ACC DIV2~AD	age	0,012	0,250	0,006	1,897	0,064
ACC DIV2~AD	AD left	0,133	0,545	0,141	0,939	0,352
ACC DIV2~AD	AD right	-0,387	-1,590	0,133	-2,902	0,006
ACC DIV2~AD	age:AD left	-0,011	-0,746	0,010	-1,202	0,235
ACC DIV2~AD	age:AD right	0,024	1,567	0,009	2,65	0,011
ACC DIV3~AD	Intercept	0,395	NA	0,149	2,661	0,010
ACC DIV3~AD	age	0,012	0,194	0,009	1,355	0,181
ACC DIV3~AD	AD left	-0,085	-0,268	0,201	-0,425	0,673
ACC DIV3~AD	AD right	0,025	0,078	0,190	0,132	0,896
ACC DIV3~AD	age:AD left	0,002	0,081	0,014	0,121	0,904
ACC DIV3~AD	age:AD right	-0,002	-0,074	0,013	-0,12	0,908
RT ADD1~AD	Intercept	2,717	NA	0,248	10,958	0,000
RT ADD1~AD	age	-0,063	-0,518	0,015	-4,215	0,000
RT ADD1~AD	AD left	-0,211	-0,341	0,335	-0,630	0,532
RT ADD1~AD	AD right	0,272	0,439	0,316	0,860	0,394

RT ADD1~AD	age:AD left	0,009	0,229	0,023	0,396	0,694
RT ADD1~AD	age:AD right	-0,010	-0,247	0,022	-0,45	0,656
RT ADD2~AD	Intercept	7,186	NA	0,739	9,718	0,000
RT ADD2~AD	age	-0,149	-0,430	0,045	-3,343	0,002
RT ADD2~AD	AD left	-0,845	-0,478	1,000	-0,844	0,402
RT ADD2~AD	AD right	0,348	0,197	0,944	0,369	0,714
RT ADD2~AD	age:AD left	0,030	0,265	0,068	0,437	0,664
RT ADD2~AD	age:AD right	0,001	0,009	0,065	0,02	0,988
RT ADD3~AD	Intercept	11,124	NA	1,387	8,020	0,000
RT ADD3~AD	age	-0,182	-0,302	0,084	-2,169	0,035
RT ADD3~AD	AD left	-0,400	-0,131	1,876	-0,213	0,832
RT ADD3~AD	AD right	-1,429	-0,466	1,770	-0,807	0,423
RT ADD3~AD	age:AD left	0,035	0,179	0,127	0,273	0,786
RT ADD3~AD	age:AD right	0,086	0,440	0,122	0,71	0,484
RT SUB1~AD	Intercept	3,391	NA	0,637	5,321	0,000
RT SUB1~AD	age	-0,081	-0,286	0,038	-2,098	0,041
RT SUB1~AD	AD left	0,011	0,007	0,862	0,012	0,990
RT SUB1~AD	AD right	0,913	0,635	0,813	1,123	0,267
RT SUB1~AD	age:AD left	0,001	0,009	0,058	0,015	0,988
RT SUB1~AD	age:AD right	-0,039	-0,427	0,056	-0,70	0,487
RT SUB2~AD	Intercept	8,141	NA	1,137	7,159	0,000
RT SUB2~AD	age	-0,129	-0,266	0,069	-1,875	0,067
RT SUB2~AD	AD left	-0,897	-0,365	1,538	-0,583	0,562
RT SUB2~AD	AD right	0,029	0,012	1,451	0,020	0,984
RT SUB2~AD	age:AD left	0,052	0,334	0,104	0,500	0,620
RT SUB2~AD	age:AD right	0,008	0,052	0,100	0,08	0,935
RT SUB3~AD	Intercept	17,705	NA	2,500	7,081	0,000
RT SUB3~AD	age	-0,350	-0,306	0,151	-2,315	0,025

RT SUB3~AD	AD left	-1,476	-0,254	3,382	-0,436	0,664
RT SUB3~AD	AD right	-4,507	-0,776	3,191	-1,412	0,164
RT SUB3~AD	age:AD left	0,057	0,156	0,228	0,251	0,803
RT SUB3~AD	age:AD right	0,218	0,590	0,219	0,99	0,325
RT MUL1~AD	Intercept	3,773	NA	0,408	9,244	0,000
RT MUL1~AD	age	-0,102	-0,506	0,025	-4,136	0,000
RT MUL1~AD	AD left	-0,786	-0,767	0,552	-1,423	0,161
RT MUL1~AD	AD right	0,895	0,874	0,521	1,718	0,092
RT MUL1~AD	age:AD left	0,042	0,648	0,037	1,125	0,266
RT MUL1~AD	age:AD right	-0,045	-0,685	0,036	-1,25	0,218
RT MUL2~AD	Intercept	12,080	NA	1,492	8,097	0,000
RT MUL2~AD	age	-0,291	-0,421	0,090	-3,226	0,002
RT MUL2~AD	AD left	-2,181	-0,621	2,018	-1,081	0,285
RT MUL2~AD	AD right	0,348	0,099	1,904	0,183	0,856
RT MUL2~AD	age:AD left	0,098	0,442	0,136	0,720	0,475
RT MUL2~AD	age:AD right	0,006	0,026	0,131	0,04	0,965
RT MUL3~AD	Intercept	21,684	NA	2,623	8,267	0,000
RT MUL3~AD	age	-0,480	-0,393	0,158	-3,028	0,004
RT MUL3~AD	AD left	-2,354	-0,380	3,548	-0,663	0,510
RT MUL3~AD	AD right	-4,036	-0,651	3,348	-1,205	0,234
RT MUL3~AD	age:AD left	0,153	0,391	0,240	0,638	0,526
RT MUL3~AD	age:AD right	0,177	0,447	0,230	0,77	0,446
RT DIV1~AD	Intercept	4,097	NA	0,761	5,384	0,000
RT DIV1~AD	age	-0,107	-0,314	0,046	-2,327	0,024
RT DIV1~AD	AD left	-0,347	-0,200	1,029	-0,337	0,738
RT DIV1~AD	AD right	1,231	0,710	0,971	1,268	0,211
RT DIV1~AD	age:AD left	0,022	0,205	0,070	0,323	0,748
RT DIV1~AD	age:AD right	-0,055	-0,494	0,067	-0,82	0,417

RT DIV2~AD	Intercept	13,289	NA	1,953	6,803	0,000
RT DIV2~AD	age	-0,305	-0,357	0,118	-2,587	0,013
RT DIV2~AD	AD left	-1,480	-0,340	2,642	-0,560	0,578
RT DIV2~AD	AD right	-0,076	-0,017	2,493	-0,030	0,976
RT DIV2~AD	age:AD left	0,082	0,299	0,178	0,460	0,647
RT DIV2~AD	age:AD right	0,013	0,048	0,171	0,08	0,939
RT DIV3~AD	Intercept	14,384	NA	2,638	5,453	0,000
RT DIV3~AD	age	-0,141	-0,124	0,159	-0,887	0,379
RT DIV3~AD	AD left	-7,531	-1,297	3,568	-2,111	0,040
RT DIV3~AD	AD right	3,531	0,608	3,367	1,049	0,299
RT DIV3~AD	age:AD left	0,422	1,151	0,241	1,751	0,086
RT DIV3~AD	age:AD right	-0,169	-0,458	0,231	-0,73	0,468
ACC ADD1~RD	Intercept	0,892	NA	0,020	45,498	0,000
ACC ADD1~RD	age	0,003	0,344	0,001	2,549	0,014
ACC ADD1~RD	RD left	0,042	0,959	0,024	1,786	0,080
ACC ADD1~RD	RD right	-0,006	-0,144	0,027	-0,236	0,814
ACC ADD1~RD	age:RD left	-0,003	-1,015	0,001	-1,933	0,059
ACC ADD1~RD	age:RD right	0,000	0,151	0,002	0,25	0,804
ACC ADD2~RD	Intercept	0,795	NA	0,055	14,539	0,000
ACC ADD2~RD	age	0,004	0,194	0,003	1,351	0,183
ACC ADD2~RD	RD left	0,101	0,871	0,066	1,522	0,134
ACC ADD2~RD	RD right	-0,093	-0,805	0,075	-1,241	0,220
ACC ADD2~RD	age:RD left	-0,006	-0,860	0,004	-1,537	0,131
ACC ADD2~RD	age:RD right	0,005	0,719	0,004	1,12	0,270
ACC ADD3~RD	Intercept	0,619	NA	0,098	6,324	0,000
ACC ADD3~RD	age	0,008	0,203	0,006	1,397	0,169
ACC ADD3~RD	RD left	0,019	0,091	0,119	0,157	0,876
ACC ADD3~RD	RD right	-0,081	-0,393	0,134	-0,600	0,551

ACC ADD3~RD	age:RD left	-0,002	-0,170	0,007	-0,302	0,764
ACC ADD3~RD	age:RD right	0,004	0,346	0,008	0,53	0,598
ACC SUB1~RD	Intercept	0,946	NA	0,022	43,248	0,000
ACC SUB1~RD	age	0,000	0,031	0,001	0,205	0,838
ACC SUB1~RD	RD left	0,005	0,105	0,027	0,177	0,861
ACC SUB1~RD	RD right	0,002	0,039	0,030	0,058	0,954
ACC SUB1~RD	age:RD left	0,000	-0,130	0,002	-0,223	0,824
ACC SUB1~RD	age:RD right	0,000	-0,079	0,002	-0,12	0,907
ACC SUB2~RD	Intercept	0,816	NA	0,065	12,594	0,000
ACC SUB2~RD	age	0,002	0,075	0,004	0,511	0,611
ACC SUB2~RD	RD left	0,008	0,058	0,079	0,100	0,921
ACC SUB2~RD	RD right	-0,066	-0,489	0,089	-0,738	0,464
ACC SUB2~RD	age:RD left	-0,001	-0,065	0,005	-0,115	0,909
ACC SUB2~RD	age:RD right	0,003	0,333	0,005	0,51	0,615
ACC SUB3~RD	Intercept	0,693	NA	0,128	5,427	0,000
ACC SUB3~RD	age	-0,002	-0,046	0,008	-0,314	0,755
ACC SUB3~RD	RD left	0,083	0,315	0,155	0,537	0,594
ACC SUB3~RD	RD right	0,033	0,125	0,176	0,188	0,852
ACC SUB3~RD	age:RD left	-0,006	-0,367	0,009	-0,640	0,525
ACC SUB3~RD	age:RD right	-0,003	-0,225	0,010	-0,34	0,735
ACC MUL1~RD	Intercept	0,915	NA	0,024	38,881	0,000
ACC MUL1~RD	age	0,002	0,251	0,001	1,749	0,086
ACC MUL1~RD	RD left	-0,008	-0,168	0,029	-0,294	0,770
ACC MUL1~RD	RD right	0,003	0,064	0,032	0,099	0,922
ACC MUL1~RD	age:RD left	0,000	0,036	0,002	0,064	0,949
ACC MUL1~RD	age:RD right	0,001	0,221	0,002	0,34	0,733
ACC MUL2~RD	Intercept	0,625	NA	0,091	6,885	0,000
ACC MUL2~RD	age	0,010	0,262	0,005	1,852	0,070

ACC MUL2~RD	RD left	0,065	0,334	0,110	0,593	0,556
ACC MUL2~RD	RD right	-0,152	-0,777	0,125	-1,217	0,229
ACC MUL2~RD	age:RD left	-0,004	-0,323	0,007	-0,587	0,560
ACC MUL2~RD	age:RD right	0,007	0,645	0,007	1,02	0,314
ACC MUL3~RD	Intercept	0,121	NA	0,130	0,932	0,356
ACC MUL3~RD	age	0,025	0,416	0,008	3,227	0,002
ACC MUL3~RD	RD left	-0,087	-0,283	0,158	-0,551	0,584
ACC MUL3~RD	RD right	0,155	0,504	0,179	0,865	0,391
ACC MUL3~RD	age:RD left	-0,002	-0,086	0,009	-0,171	0,865
ACC MUL3~RD	age:RD right	-0,004	-0,208	0,010	-0,36	0,721
ACC DIV1~RD	Intercept	0,845	NA	0,050	16,999	0,000
ACC DIV1~RD	age	0,005	0,227	0,003	1,548	0,128
ACC DIV1~RD	RD left	0,035	0,343	0,060	0,588	0,559
ACC DIV1~RD	RD right	-0,032	-0,313	0,068	-0,472	0,639
ACC DIV1~RD	age:RD left	-0,002	-0,288	0,004	-0,504	0,616
ACC DIV1~RD	age:RD right	0,002	0,379	0,004	0,58	0,567
ACC DIV2~RD	Intercept	0,633	NA	0,112	5,668	0,000
ACC DIV2~RD	age	0,010	0,217	0,007	1,554	0,127
ACC DIV2~RD	RD left	0,163	0,669	0,135	1,200	0,236
ACC DIV2~RD	RD right	-0,298	-1,224	0,154	-1,937	0,058
ACC DIV2~RD	age:RD left	-0,012	-0,817	0,008	-1,500	0,140
ACC DIV2~RD	age:RD right	0,018	1,269	0,009	2,02	0,049
ACC DIV3~RD	Intercept	0,414	NA	0,151	2,740	0,008
ACC DIV3~RD	age	0,011	0,178	0,009	1,232	0,224
ACC DIV3~RD	RD left	-0,050	-0,155	0,183	-0,270	0,788
ACC DIV3~RD	RD right	-0,009	-0,027	0,208	-0,042	0,967
ACC DIV3~RD	age:RD left	-0,002	-0,096	0,011	-0,171	0,865
ACC DIV3~RD	age:RD right	0,003	0,159	0,012	0,25	0,807

RT ADD1~RD	Intercept	2,762	NA	0,258	10,711	0,000
RT ADD1~RD	age	-0,066	-0,537	0,015	-4,242	0,000
RT ADD1~RD	RD left	-0,086	-0,139	0,313	-0,276	0,784
RT ADD1~RD	RD right	0,098	0,158	0,355	0,276	0,784
RT ADD1~RD	age:RD left	0,008	0,200	0,019	0,406	0,687
RT ADD1~RD	age:RD right	-0,007	-0,190	0,020	-0,33	0,740
RT ADD2~RD	Intercept	7,129	NA	0,766	9,310	0,000
RT ADD2~RD	age	-0,145	-0,418	0,046	-3,165	0,003
RT ADD2~RD	RD left	0,402	0,228	0,929	0,433	0,667
RT ADD2~RD	RD right	-0,246	-0,139	1,053	-0,233	0,816
RT ADD2~RD	age:RD left	-0,010	-0,091	0,055	-0,176	0,861
RT ADD2~RD	age:RD right	0,015	0,148	0,060	0,25	0,803
RT ADD3~RD	Intercept	10,424	NA	1,364	7,644	0,000
RT ADD3~RD	age	-0,139	-0,231	0,082	-1,703	0,095
RT ADD3~RD	RD left	2,474	0,807	1,654	1,496	0,141
RT ADD3~RD	RD right	-2,148	-0,701	1,875	-1,145	0,257
RT ADD3~RD	age:RD left	-0,089	-0,477	0,098	-0,904	0,370
RT ADD3~RD	age:RD right	0,109	0,623	0,107	1,02	0,311
RT SUB1~RD	Intercept	3,486	NA	0,673	5,182	0,000
RT SUB1~RD	age	-0,085	-0,299	0,040	-2,097	0,041
RT SUB1~RD	RD left	0,301	0,210	0,816	0,369	0,713
RT SUB1~RD	RD right	-0,387	-0,269	0,925	-0,418	0,677
RT SUB1~RD	age:RD left	-0,007	-0,083	0,048	-0,150	0,881
RT SUB1~RD	age:RD right	0,012	0,150	0,053	0,23	0,816
RT SUB2~RD	Intercept	8,094	NA	1,150	7,037	0,000
RT SUB2~RD	age	-0,120	-0,249	0,069	-1,748	0,087
RT SUB2~RD	RD left	0,460	0,187	1,395	0,330	0,743
RT SUB2~RD	RD right	-1,479	-0,601	1,582	-0,935	0,354

RT SUB2~RD	age:RD left	-0,004	-0,029	0,083	-0,053	0,958
RT SUB2~RD	age:RD right	0,072	0,515	0,090	0,81	0,424
RT SUB3~RD	Intercept	16,919	NA	2,626	6,443	0,000
RT SUB3~RD	age	-0,299	-0,262	0,157	-1,902	0,063
RT SUB3~RD	RD left	4,883	0,840	3,184	1,534	0,131
RT SUB3~RD	RD right	-6,597	-1,135	3,611	-1,827	0,074
RT SUB3~RD	age:RD left	-0,218	-0,616	0,189	-1,151	0,255
RT SUB3~RD	age:RD right	0,316	0,952	0,205	1,54	0,130
RT MUL1~RD	Intercept	3,876	NA	0,432	8,972	0,000
RT MUL1~RD	age	-0,106	-0,526	0,026	-4,098	0,000
RT MUL1~RD	RD left	-0,533	-0,521	0,524	-1,018	0,314
RT MUL1~RD	RD right	0,407	0,397	0,594	0,685	0,497
RT MUL1~RD	age:RD left	0,032	0,509	0,031	1,018	0,313
RT MUL1~RD	age:RD right	-0,023	-0,396	0,034	-0,69	0,495
RT MUL2~RD	Intercept	11,835	NA	1,532	7,724	0,000
RT MUL2~RD	age	-0,271	-0,392	0,092	-2,954	0,005
RT MUL2~RD	RD left	1,181	0,336	1,858	0,636	0,528
RT MUL2~RD	RD right	-1,532	-0,436	2,107	-0,727	0,471
RT MUL2~RD	age:RD left	-0,029	-0,133	0,110	-0,258	0,797
RT MUL2~RD	age:RD right	0,077	0,381	0,120	0,64	0,525
RT MUL3~RD	Intercept	20,814	NA	2,683	7,757	0,000
RT MUL3~RD	age	-0,422	-0,346	0,161	-2,627	0,011
RT MUL3~RD	RD left	4,720	0,761	3,254	1,451	0,153
RT MUL3~RD	RD right	-6,376	-1,028	3,690	-1,728	0,090
RT MUL3~RD	age:RD left	-0,164	-0,436	0,193	-0,850	0,399
RT MUL3~RD	age:RD right	0,270	0,761	0,210	1,29	0,204
RT DIV1~RD	Intercept	4,386	NA	0,803	5,460	0,000
RT DIV1~RD	age	-0,121	-0,354	0,048	-2,511	0,015

RT DIV1~RD	RD left	-0,599	-0,345	0,974	-0,615	0,541
RT DIV1~RD	RD right	0,064	0,037	1,105	0,058	0,954
RT DIV1~RD	age:RD left	0,037	0,354	0,058	0,645	0,522
RT DIV1~RD	age:RD right	-0,011	-0,112	0,063	-0,18	0,860
RT DIV2~RD	Intercept	12,728	NA	1,957	6,505	0,000
RT DIV2~RD	age	-0,273	-0,319	0,117	-2,328	0,024
RT DIV2~RD	RD left	0,783	0,180	2,373	0,330	0,743
RT DIV2~RD	RD right	0,496	0,114	2,691	0,184	0,854
RT DIV2~RD	age:RD left	0,008	0,030	0,141	0,056	0,956
RT DIV2~RD	age:RD right	-0,032	-0,131	0,153	-0,21	0,833
RT DIV3~RD	Intercept	16,313	NA	2,592	6,293	0,000
RT DIV3~RD	age	-0,244	-0,214	0,155	-1,574	0,122
RT DIV3~RD	RD left	-9,344	-1,610	3,144	-2,972	0,005
RT DIV3~RD	RD right	5,579	0,961	3,565	1,565	0,124
RT DIV3~RD	age:RD left	0,476	1,348	0,187	2,547	0,014
RT DIV3~RD	age:RD right	-0,302	-0,910	0,203	-1,49	0,142
ACC ADD1~MD	Intercept	0,896	NA	0,020	45,807	0,000
ACC ADD1~MD	age	0,003	0,321	0,001	2,380	0,021
ACC ADD1~MD	MD left	0,040	0,901	0,024	1,676	0,100
ACC ADD1~MD	MD right	-0,012	-0,267	0,024	-0,487	0,629
ACC ADD1~MD	age:MD left	-0,003	-0,920	0,001	-1,699	0,096
ACC ADD1~MD	age:MD right	0,000	0,164	0,001	0,29	0,771
ACC ADD2~MD	Intercept	0,803	NA	0,054	14,951	0,000
ACC ADD2~MD	age	0,004	0,181	0,003	1,281	0,206
ACC ADD2~MD	MD left	0,093	0,807	0,065	1,430	0,159
ACC ADD2~MD	MD right	-0,113	-0,975	0,067	-1,692	0,097
ACC ADD2~MD	age:MD left	-0,006	-0,791	0,004	-1,391	0,170
ACC ADD2~MD	age:MD right	0,006	0,818	0,004	1,39	0,171

ACC ADD3~MD	Intercept	0,618	NA	0,094	6,544	0,000
ACC ADD3~MD	age	0,008	0,209	0,006	1,491	0,142
ACC ADD3~MD	MD left	0,111	0,541	0,115	0,967	0,338
ACC ADD3~MD	MD right	-0,197	-0,959	0,117	-1,677	0,100
ACC ADD3~MD	age:MD left	-0,006	-0,477	0,007	-0,846	0,401
ACC ADD3~MD	age:MD right	0,009	0,748	0,007	1,28	0,206
ACC SUB1~MD	Intercept	0,950	NA	0,021	44,909	0,000
ACC SUB1~MD	age	0,000	0,008	0,001	0,053	0,958
ACC SUB1~MD	MD left	0,017	0,389	0,026	0,671	0,505
ACC SUB1~MD	MD right	-0,023	-0,521	0,026	-0,881	0,382
ACC SUB1~MD	age:MD left	-0,001	-0,399	0,002	-0,683	0,498
ACC SUB1~MD	age:MD right	0,001	0,312	0,002	0,52	0,608
ACC SUB2~MD	Intercept	0,826	NA	0,061	13,628	0,000
ACC SUB2~MD	age	0,002	0,057	0,004	0,417	0,678
ACC SUB2~MD	MD left	0,036	0,269	0,074	0,489	0,627
ACC SUB2~MD	MD right	-0,140	-1,042	0,075	-1,857	0,069
ACC SUB2~MD	age:MD left	-0,001	-0,141	0,005	-0,256	0,799
ACC SUB2~MD	age:MD right	0,005	0,668	0,005	1,17	0,249
ACC SUB3~MD	Intercept	0,698	NA	0,126	5,522	0,000
ACC SUB3~MD	age	-0,002	-0,041	0,008	-0,278	0,782
ACC SUB3~MD	MD left	0,143	0,540	0,154	0,927	0,358
ACC SUB3~MD	MD right	-0,107	-0,407	0,157	-0,684	0,497
ACC SUB3~MD	age:MD left	-0,007	-0,461	0,010	-0,785	0,436
ACC SUB3~MD	age:MD right	0,003	0,186	0,009	0,31	0,761
ACC MUL1~MD	Intercept	0,916	NA	0,024	38,665	0,000
ACC MUL1~MD	age	0,002	0,246	0,001	1,703	0,095
ACC MUL1~MD	MD left	0,004	0,088	0,029	0,152	0,880
ACC MUL1~MD	MD right	-0,015	-0,294	0,029	-0,498	0,621

ACC MUL1~MD	age:MD left	-0,001	-0,210	0,002	-0,361	0,720
ACC MUL1~MD	age:MD right	0,001	0,480	0,002	0,80	0,429
ACC MUL2~MD	Intercept	0,635	NA	0,087	7,342	0,000
ACC MUL2~MD	age	0,010	0,250	0,005	1,847	0,071
ACC MUL2~MD	MD left	0,102	0,521	0,105	0,967	0,338
ACC MUL2~MD	MD right	-0,218	-1,116	0,108	-2,026	0,048
ACC MUL2~MD	age:MD left	-0,006	-0,478	0,007	-0,880	0,383
ACC MUL2~MD	age:MD right	0,010	0,860	0,006	1,53	0,132
ACC MUL3~MD	Intercept	0,098	NA	0,132	0,738	0,464
ACC MUL3~MD	age	0,027	0,442	0,008	3,362	0,001
ACC MUL3~MD	MD left	0,002	0,008	0,161	0,015	0,988
ACC MUL3~MD	MD right	0,084	0,272	0,165	0,507	0,614
ACC MUL3~MD	age:MD left	-0,005	-0,278	0,010	-0,527	0,601
ACC MUL3~MD	age:MD right	-0,001	-0,047	0,010	-0,09	0,932
ACC DIV1~MD	Intercept	0,850	NA	0,048	17,707	0,000
ACC DIV1~MD	age	0,004	0,222	0,003	1,558	0,125
ACC DIV1~MD	MD left	0,078	0,755	0,058	1,332	0,189
ACC DIV1~MD	MD right	-0,114	-1,109	0,060	-1,916	0,061
ACC DIV1~MD	age:MD left	-0,004	-0,667	0,004	-1,168	0,248
ACC DIV1~MD	age:MD right	0,006	1,045	0,004	1,77	0,083
ACC DIV2~MD	Intercept	0,630	NA	0,106	5,937	0,000
ACC DIV2~MD	age	0,011	0,231	0,006	1,730	0,090
ACC DIV2~MD	MD left	0,196	0,808	0,129	1,520	0,135
ACC DIV2~MD	MD right	-0,385	-1,581	0,132	-2,912	0,005
ACC DIV2~MD	age:MD left	-0,014	-0,962	0,008	-1,796	0,079
ACC DIV2~MD	age:MD right	0,022	1,571	0,008	2,84	0,007
ACC DIV3~MD	Intercept	0,411	NA	0,150	2,740	0,008
ACC DIV3~MD	age	0,011	0,181	0,009	1,263	0,213

ACC DIV3~MD	MD left	-0,062	-0,195	0,183	-0,341	0,735
ACC DIV3~MD	MD right	-0,005	-0,015	0,186	-0,025	0,980
ACC DIV3~MD	age:MD left	-0,001	-0,059	0,011	-0,102	0,919
ACC DIV3~MD	age:MD right	0,002	0,100	0,011	0,17	0,867
RT ADD1~MD	Intercept	2,752	NA	0,254	10,819	0,000
RT ADD1~MD	age	-0,065	-0,536	0,015	-4,275	0,000
RT ADD1~MD	MD left	-0,221	-0,356	0,310	-0,712	0,479
RT ADD1~MD	MD right	0,284	0,459	0,316	0,899	0,373
RT ADD1~MD	age:MD left	0,013	0,336	0,019	0,668	0,508
RT ADD1~MD	age:MD right	-0,014	-0,383	0,019	-0,74	0,465
RT ADD2~MD	Intercept	7,230	NA	0,765	9,446	0,000
RT ADD2~MD	age	-0,152	-0,437	0,046	-3,298	0,002
RT ADD2~MD	MD left	-0,254	-0,144	0,932	-0,273	0,786
RT ADD2~MD	MD right	0,257	0,145	0,952	0,269	0,789
RT ADD2~MD	age:MD left	0,015	0,135	0,058	0,253	0,801
RT ADD2~MD	age:MD right	-0,005	-0,044	0,057	-0,08	0,937
RT ADD3~MD	Intercept	10,810	NA	1,382	7,824	0,000
RT ADD3~MD	age	-0,160	-0,266	0,083	-1,929	0,059
RT ADD3~MD	MD left	1,641	0,535	1,682	0,975	0,334
RT ADD3~MD	MD right	-2,029	-0,662	1,719	-1,180	0,243
RT ADD3~MD	age:MD left	-0,055	-0,290	0,104	-0,524	0,603
RT ADD3~MD	age:MD right	0,107	0,595	0,103	1,04	0,303
RT SUB1~MD	Intercept	3,418	NA	0,666	5,130	0,000
RT SUB1~MD	age	-0,082	-0,292	0,040	-2,060	0,045
RT SUB1~MD	MD left	-0,062	-0,043	0,811	-0,076	0,939
RT SUB1~MD	MD right	0,529	0,368	0,829	0,638	0,526
RT SUB1~MD	age:MD left	0,009	0,105	0,050	0,184	0,854
RT SUB1~MD	age:MD right	-0,027	-0,325	0,050	-0,55	0,583

RT SUB2~MD	Intercept	8,169	NA	1,152	7,093	0,000
RT SUB2~MD	age	-0,127	-0,262	0,069	-1,835	0,072
RT SUB2~MD	MD left	-0,197	-0,080	1,402	-0,140	0,889
RT SUB2~MD	MD right	-0,624	-0,254	1,433	-0,436	0,665
RT SUB2~MD	age:MD left	0,024	0,160	0,087	0,279	0,781
RT SUB2~MD	age:MD right	0,034	0,232	0,086	0,39	0,697
RT SUB3~MD	Intercept	17,801	NA	2,575	6,912	0,000
RT SUB3~MD	age	-0,348	-0,305	0,155	-2,249	0,029
RT SUB3~MD	MD left	3,183	0,548	3,136	1,015	0,315
RT SUB3~MD	MD right	-6,629	-1,141	3,204	-2,069	0,044
RT SUB3~MD	age:MD left	-0,151	-0,423	0,194	-0,779	0,440
RT SUB3~MD	age:MD right	0,308	0,903	0,192	1,61	0,115
RT MUL1~MD	Intercept	3,847	NA	0,420	9,166	0,000
RT MUL1~MD	age	-0,106	-0,525	0,025	-4,193	0,000
RT MUL1~MD	MD left	-0,823	-0,803	0,511	-1,610	0,114
RT MUL1~MD	MD right	0,842	0,822	0,522	1,613	0,113
RT MUL1~MD	age:MD left	0,045	0,719	0,032	1,431	0,159
RT MUL1~MD	age:MD right	-0,043	-0,717	0,031	-1,38	0,174
RT MUL2~MD	Intercept	12,151	NA	1,545	7,866	0,000
RT MUL2~MD	age	-0,292	-0,422	0,093	-3,141	0,003
RT MUL2~MD	MD left	-0,363	-0,103	1,881	-0,193	0,848
RT MUL2~MD	MD right	-0,370	-0,105	1,922	-0,192	0,848
RT MUL2~MD	age:MD left	0,033	0,153	0,116	0,283	0,778
RT MUL2~MD	age:MD right	0,026	0,124	0,115	0,22	0,825
RT MUL3~MD	Intercept	21,734	NA	2,657	8,179	0,000
RT MUL3~MD	age	-0,474	-0,389	0,160	-2,968	0,005
RT MUL3~MD	MD left	2,733	0,441	3,236	0,845	0,402
RT MUL3~MD	MD right	-6,213	-1,002	3,306	-1,879	0,066

RT MUL3~MD	age:MD left	-0,078	-0,204	0,200	-0,388	0,700
RT MUL3~MD	age:MD right	0,257	0,706	0,198	1,30	0,200
RT DIV1~MD	Intercept	4,216	NA	0,794	5,312	0,000
RT DIV1~MD	age	-0,114	-0,333	0,048	-2,382	0,021
RT DIV1~MD	MD left	-0,851	-0,491	0,966	-0,881	0,383
RT DIV1~MD	MD right	1,021	0,589	0,987	1,034	0,306
RT DIV1~MD	age:MD left	0,050	0,471	0,060	0,838	0,406
RT DIV1~MD	age:MD right	-0,052	-0,513	0,059	-0,88	0,382
RT DIV2~MD	Intercept	13,129	NA	1,974	6,650	0,000
RT DIV2~MD	age	-0,295	-0,345	0,119	-2,488	0,016
RT DIV2~MD	MD left	-0,070	-0,016	2,404	-0,029	0,977
RT DIV2~MD	MD right	0,274	0,063	2,456	0,111	0,912
RT DIV2~MD	age:MD left	0,039	0,144	0,149	0,260	0,796
RT DIV2~MD	age:MD right	-0,017	-0,067	0,147	-0,12	0,908
RT DIV3~MD	Intercept	15,556	NA	2,568	6,059	0,000
RT DIV3~MD	age	-0,205	-0,180	0,154	-1,328	0,190
RT DIV3~MD	MD left	-9,576	-1,650	3,126	-3,063	0,004
RT DIV3~MD	MD right	5,632	0,970	3,194	1,763	0,084
RT DIV3~MD	age:MD left	0,501	1,404	0,194	2,588	0,013
RT DIV3~MD	age:MD right	-0,293	-0,860	0,191	-1,53	0,132

ACC – accuracy, RT – reaction time, ADD – addition, SUB – subtraction, MUL – multiplication, DIV – division, FA – fractional anisotropy, AD – axial diffusivity, RD – radial diffusivity, MD – mean diffusivity. 1, 2, and 3 correspond to difficulty levels 1, 2, and 3, respectively. Model: PMT score ~ age(DTI left MdLF + DTI right MdLF).*
