# Supplementary material for: Math skills and microstructure of the middle longitudinal fasciculus: A developmental investigation
Source: PLoS One. 2025 Jun 11;20(6):e0324802. doi: 10.1371/journal.pone.0324802 (PMC12157344; doi:10.1371/journal.pone.0324802)
Supplement: S1 File — S1 Table. PMT performance scores (accuracy and reaction time) as a function of age group and across three levels of difficulty for addition, subtraction, multiplication, and division tasks. S2 Table. DTI metrics (FA, AD, RD, and MD) in bilateral MdLF, as a function of age group. S3 Table. Pearson r and p-values for the correlations among DTI metrics (FA, AD, RD, and MD) in bilateral MdLF. S4 Table. Descriptive statistics (mean, SD) for PMT accuracy as a function of age group and across three levels of difficulty for addition, subtraction, multiplication, and division tasks. S5 Table. Descriptive statistics (mean, SD) for PMT reaction time as a function of age group and across three levels of difficulty for addition, subtraction, multiplication, and division tasks. S6 Table. Results of ANOVA analyses for PMT accuracy across three levels of difficulty for addition, subtraction, multiplication, and division tasks in children, adolescents, and adults. S7 Table. Results of ANOVA analyses for PMT reaction time across three levels of difficulty for addition, subtraction, multiplication, and division tasks in children, adolescents, and adults. S8 Table. Descriptive statistics (mean, SD) for DTI metrics (FA, AD, RD, and MD) in children, adolescents, and adults. S9 Table. Results of ANOVA analyses for DTI metrics (FA, AD, RD, and MD) in children, adolescents, and adults. S10 Table. Table Results of between-group comparison (t-test) for DTI metrics in the left and right MdLF. S11 Table. Results of linear regression models for each PMT performance score (accuracy, reaction time) and DTI metric (FA, AD, RD, and MD). S12 Table. Results of linear regression models for each PMT performance score (accuracy, reaction time) and DTI metric (FA, AD, RD, and MD) – model statistics. S13 Table. Pearson r and p-values for the correlations between PMT accuracy and DTI metric (FA, AD, RD, and MD) before and after controlling for age. S14 Table. Pearson r and p-values for the correlations betw [file pone.0324802.s001.zip › Supporting information_update210525/S4 Table.Descriptive statistics (mean, SD) for PMT accuracy across three levels of addition, subtraction, multiplication, and division tasks in children, adolescents, and adults.pdf]

S4 Table. Descriptive statistics (mean, SD) for PMT accuracy across three levels of addition, subtraction, multiplication, and division tasks in children, adolescents, and adults

	Accuracy							
	Addition Level 1		Subtraction Level 1		Multiplication Level 1		Division Level 1	
	Mean	SD	Mean	SD	Mean	SD	Mean	SD
**Children**	0,922	0,057	0,950	0,053	0,935	0,064	0,895	0,147
**Adolescents**	0,951	0,028	0,946	0,043	0,971	0,034	0,931	0,068
**Adults**	0,963	0,027	0,959	0,033	0,958	0,035	0,937	0,043
	**Addition Level 2**		**Subtraction Level 2**		**Multiplication Level 2**		**Division Level 2**	
	**Mean**	**SD**	**Mean**	**SD**	**Mean**	**SD**	**Mean**	**SD**
**Children**	0,855	0,130	0,828	0,171	0,717	0,244	0,743	0,281
**Adolescents**	0,845	0,121	0,854	0,120	0,795	0,166	0,787	0,215
**Adults**	0,916	0,067	0,859	0,085	0,865	0,101	0,871	0,210
	**Addition Level 3**		**Subtraction Level 3**		**Multiplication Level 3**		**Division Level 3**	
	**Mean**	**SD**	**Mean**	**SD**	**Mean**	**SD**	**Mean**	**SD**
**Children**	0,697	0,244	0,690	0,310	0,365	0,318	0,514	0,380
**Adolescents**	0,756	0,200	0,622	0,250	0,588	0,279	0,633	0,274
**Adults**	0,814	0,120	0,692	0,208	0,720	0,182	0,656	0,264

SD – standard deviation.
