# Supplementary material for: Math skills and microstructure of the middle longitudinal fasciculus: A developmental investigation
Source: PLoS One. 2025 Jun 11;20(6):e0324802. doi: 10.1371/journal.pone.0324802 (PMC12157344; doi:10.1371/journal.pone.0324802)
Supplement: S1 File — S1 Table. PMT performance scores (accuracy and reaction time) as a function of age group and across three levels of difficulty for addition, subtraction, multiplication, and division tasks. S2 Table. DTI metrics (FA, AD, RD, and MD) in bilateral MdLF, as a function of age group. S3 Table. Pearson r and p-values for the correlations among DTI metrics (FA, AD, RD, and MD) in bilateral MdLF. S4 Table. Descriptive statistics (mean, SD) for PMT accuracy as a function of age group and across three levels of difficulty for addition, subtraction, multiplication, and division tasks. S5 Table. Descriptive statistics (mean, SD) for PMT reaction time as a function of age group and across three levels of difficulty for addition, subtraction, multiplication, and division tasks. S6 Table. Results of ANOVA analyses for PMT accuracy across three levels of difficulty for addition, subtraction, multiplication, and division tasks in children, adolescents, and adults. S7 Table. Results of ANOVA analyses for PMT reaction time across three levels of difficulty for addition, subtraction, multiplication, and division tasks in children, adolescents, and adults. S8 Table. Descriptive statistics (mean, SD) for DTI metrics (FA, AD, RD, and MD) in children, adolescents, and adults. S9 Table. Results of ANOVA analyses for DTI metrics (FA, AD, RD, and MD) in children, adolescents, and adults. S10 Table. Table Results of between-group comparison (t-test) for DTI metrics in the left and right MdLF. S11 Table. Results of linear regression models for each PMT performance score (accuracy, reaction time) and DTI metric (FA, AD, RD, and MD). S12 Table. Results of linear regression models for each PMT performance score (accuracy, reaction time) and DTI metric (FA, AD, RD, and MD) – model statistics. S13 Table. Pearson r and p-values for the correlations between PMT accuracy and DTI metric (FA, AD, RD, and MD) before and after controlling for age. S14 Table. Pearson r and p-values for the correlations betw [file pone.0324802.s001.zip › Supporting information_update210525/S3 Table.Pearson r and p-values for the correlations btween DTI metrics (FA, AD, RD, and MD) in bilateral MdLF.pdf]

S3 Table. Pearson *r* and *p*-values for the correlations btween DTI metrics (FA, AD, RD, and MD) in bilateral MdLF

DTI metric 1	DTI metric 2	Pearson *r*	*p*-value
FA left MdLF	FA right MdLF	0,62	**0,000**
FA left MdLF	MD left MdLF	-0,32	**0,018**
FA left MdLF	MD right MdLF	-0,27	**0,041**
FA left MdLF	RD left MdLF	-0,65	**0,000**
FA left MdLF	RD right MdLF	-0,54	**0,000**
FA left MdLF	AD left MdLF	0,29	**0,030**
FA left MdLF	AD right MdLF	0,16	0,231
FA right MdLF	FA left MdLF	0,62	**0,000**
FA right MdLF	MD left MdLF	-0,02	0,891
FA right MdLF	MD right MdLF	-0,12	0,385
FA right MdLF	RD left MdLF	-0,25	0,061
FA right MdLF	RD right MdLF	-0,58	**0,000**
FA right MdLF	AD left MdLF	0,35	**0,009**
FA right MdLF	AD right MdLF	0,51	**0,000**
AD left MdLF	FA left MdLF	0,29	**0,030**
AD left MdLF	FA right MdLF	0,35	**0,009**
AD left MdLF	MD left MdLF	0,81	**0,000**
AD left MdLF	MD right MdLF	0,46	**0,000**
AD left MdLF	RD left MdLF	0,53	**0,000**
AD left MdLF	RD right MdLF	0,20	0,142
AD left MdLF	AD right MdLF	0,63	**0,000**
AD right MdLF	FA left MdLF	0,16	0,231
AD right MdLF	FA right MdLF	0,51	**0,000**
AD right MdLF	MD left MdLF	0,54	**0,000**
AD right MdLF	MD right MdLF	0,79	**0,000**
AD right MdLF	RD left MdLF	0,38	**0,004**
AD right MdLF	RD right MdLF	0,40	**0,002**
AD right MdLF	AD left MdLF	0,63	**0,000**
RD left MdLF	FA left MdLF	-0,65	**0,000**
RD left MdLF	FA right MdLF	-0,25	0,061
RD left MdLF	MD left MdLF	0,93	**0,000**
RD left MdLF	MD right MdLF	0,63	**0,000**
RD left MdLF	RD right MdLF	0,64	**0,000**
RD left MdLF	AD left MdLF	0,53	**0,000**
RD left MdLF	AD right MdLF	0,38	**0,004**
RD right MdLF	FA left MdLF	-0,54	**0,000**
RD right MdLF	FA right MdLF	-0,58	**0,000**
RD right MdLF	MD left MdLF	0,53	**0,000**
RD right MdLF	MD right MdLF	0,88	**0,000**
RD right MdLF	RD left MdLF	0,64	**0,000**
RD right MdLF	AD left MdLF	0,20	0,142
RD right MdLF	AD right MdLF	0,40	**0,002**
MD left MdLF	FA left MdLF	-0,32	**0,018**
MD left MdLF	FA right MdLF	-0,02	0,891
MD left MdLF	MD right MdLF	0,64	**0,000**
MD left MdLF	RD left MdLF	0,93	**0,000**
MD left MdLF	RD right MdLF	0,53	**0,000**
MD left MdLF	AD left MdLF	0,81	**0,000**
MD left MdLF	AD right MdLF	0,54	**0,000**
MD right MdLF	FA left MdLF	-0,27	**0,041**
MD right MdLF	FA right MdLF	-0,12	0,385
MD right MdLF	MD left MdLF	0,64	**0,000**
MD right MdLF	RD left MdLF	0,63	**0,000**
MD right MdLF	RD right MdLF	0,88	**0,000**
MD right MdLF	AD left MdLF	0,46	**0,000**
MD right MdLF	AD right MdLF	0,79	**0,000**

FA – fractional anisotropy, AD – axial diffusivity, RD – radial diffusivity, MD – mean diffusivity, MdLF – middle longitudinal fasciculus. Age groups 1, 2, and 3 correspond to children, adolescents, and adults, respectively.
